# An innovative NRF2 nano-modulator induces lung cancer ferroptosis and elicits an immunostimulatory tumor microenvironment

**DOI:** 10.7150/thno.57803

**Published:** 2021-05-13

**Authors:** Chih-Hsiung Hsieh, Hung-Chia Hsieh, Fu-Hsuan Shih, Pei-Wen Wang, Li-Xing Yang, Dar-Bin Shieh, Yi-Ching Wang

**Affiliations:** 1 Department of Pharmacology, National Cheng Kung University, Tainan, 70101, Taiwan.; 2 Institute of Oral Medicine, National Cheng Kung University, Tainan, 70101, Taiwan.; 3 Institute of Basic Medicine, National Cheng Kung University, Tainan, 70101, Taiwan.; 4 Center of Applied Nanomedicine, National Cheng Kung University, Tainan, 70101, Taiwan.; 5 Core Facility Center, National Cheng Kung University, Tainan 701401, Taiwan.; 6 Department of Stomatology, National Cheng Kung University Hospital, Tainan, 704302, Taiwan.

**Keywords:** nanoparticle, NRF2, ferroptosis, tumor microenvironment, lung cancer

## Abstract

Simultaneous targeting of both the tumor microenvironment and cancer cells by a single nanomedicine has not been reported to date. Here, we report the dual properties of zero-valent-iron nanoparticle (ZVI-NP) to induce cancer-specific cytotoxicity and anti-cancer immunity.

**Methods:** Cancer-specific cytotoxicity induced by ZVI-NP was determined by MTT assay. Mitochondria functional assay, immunofluorescence staining, Western blot, RT-qPCR, and ChIP-qPCR assays were used to dissect the mechanism underlying ZVI-NP-induced ferroptotic cancer cell death. The therapeutic potential of ZVI-NP was evaluated in immunocompetent mice and humanized mice. Immune cell profiles of allografts and *ex vivo* cultured immune cells were examined by flow cytometry analysis, RT-qPCR assay, and immunofluorescence.

**Results:** ZVI-NP caused mitochondria dysfunction, intracellular oxidative stress, and lipid peroxidation, leading to ferroptotic death of lung cancer cells. Degradation of NRF2 by GSK3/β-TrCP through AMPK/mTOR activation was enhanced in such cancer-specific ferroptosis. In addition, ZVI-NP attenuated self-renewal ability of cancer and downregulated angiogenesis-related genes. Importantly, ZVI-NP augmented anti-tumor immunity by shifting pro-tumor M2 macrophages to anti-tumor M1, decreasing the population of regulatory T cells, downregulating PD-1 and CTLA4 in CD8^+^ T cells to potentiate their cytolytic activity against cancer cells, while attenuating PD-L1 expression in cancer cells* in vitro* and in tumor-bearing immunocompetent mice. In particular, ZVI-NPs preferentially accumulated in tumor and lung tissues, leading to prominent suppression of tumor growth and metastasis.

**Conclusions:** This dual-functional nanomedicine established an effective strategy to synergistically induce ferroptotic cancer cell death and reprogram the immunosuppressive microenvironment, which highlights the potential of ZVI-NP as an advanced integrated anti-cancer strategy.

## Introduction

Zero-valent-iron (ZVI) nanoparticle (NP) has been extensively developed for treatment of groundwater or wastewater contaminated with various organic and inorganic pollutants due to its strong reductive potentials and wide availability 1. ZVI-NP can generate massive reactive oxygen species (ROS) through Fenton reaction and other chemical processes 2. In spite of numerous attentions on environmental remediation, there is less research on anti-tumor efficacy both in cancer cells *per se* and in tumor microenvironment (TME) immune modulation in both *in vitro* and *in vivo* studies. Previously, we discovered that silver coated ZVI-NP (ZVI@Ag) can be rapidly converted into iron ions by the enhanced lysosomal function of cancer cells 3. ZVI-NP was rapidly converted to iron ions preferentially in lysosomes of cancer cells but not in those of normal cells due to their more acidic intra-organelle compartment in cancerous state. The burst release of iron ions further induced ROS surge in cancer cells and damaged subcellular organelles leading to ferroptosis 3. In addition, the carboxymethylcellulose coated ZVI-NP (ZVI@CMC) having the biocompatible organic shell 4 can induce lipid peroxidation and trigger ferroptosis in cancer cells 5. Nevertheless, the underlying molecular mechanism of ZVI-NP-induced ferroptosis and the effect of ZVI-NP on tumor-associated immune cells remain elusive.

Ferroptosis is a novel programmed cell death identified in recent years. It is characterized by the intracellular iron accumulation and lipid peroxidation during the cell death process 6. Ferroptotic cells are morphologically characterized by small mitochondria with membrane rupture and vanishing of the crista 7, 8, which are obviously different from necrosis, apoptosis, and autophagy. Increasing evidence has shown the great potential of triggering ferroptosis as an effective anti-cancer therapy to eradicate malignancies 9, 10. For example, sorafenib, an FDA-approved anti-cancer drug, was identified as a ferroptosis inducer by blocking the synthesis of glutathione (GSH) 11. However, sorafenib may cause serious clinical adverse effects, including life-threatening cardiovascular events 12, 13. Therefore, developing anti-cancer strategies through inducing ferroptosis with higher efficacy while improving safety is of great importance.

Cancer immunotherapy through enhancing patient's own immune system to eliminate cancer cells has revolutionized the landscape of cancer therapeutics over the past decade. However, substantial benefit of immunotherapy is observed only for a limited fraction of cancer patients 14-16. Recently, several NPs have been reported to elicit tumor microenvironment modulation, such as the macrophage reprogramming by manganese dioxide NPs 17 or by magnetic NPs 18, 19, and the promotion of anti-tumor cytotoxic T cells' function by granzyme B NPs 20, as well as the augmented cytotoxic T cells recruitment to tumor site by antibody-targeted NPs 21. Moreover, the expression of pro-phagocytic calreticulin, functioning as an “eat me” signal, can be amplified on cancer cells by manganese-based NPs 22. Nevertheless, biocompatibility and large-scale production still remained the major challenges in anti-cancer nanomedicine development. Accordingly, ZVI-NP, efficiently delivering massive iron to cancer cells, might serve as a promising strategy to induce tumor ferroptosis. Besides, how ZVI-NP modulates immune responses is also worthy of further exploration.

In this study, we evaluated the potential roles of our newly developed ZVI-NP in modulating tumor microenvironment in lung cancer models and dissected the molecular mechanisms involved in ferroptosis induction. We identified a novel mechanism by which ZVI-NP enhanced phosphorylation-dependent ubiquitination and degradation of nuclear factor-E2-related factor 2 (NRF2), a pivotal transcription regulator of cellular redox homeostasis, and thereby triggering ferroptotic cell death through excessive oxidative stress and lipid peroxidation. In addition, ZVI-NP attenuated self-renewal ability of cancer cells and suppressed angiogenesis of endothelial cells. Of note, our results demonstrated that ZVI-NP reprogrammed the polarization of tumor-associated macrophages toward anti-tumor M1 phenotype and increased cytotoxic function of CD8^+^ T cells as well as reduced regulatory T cell proportion to augment anti-tumor immunity in our *ex vivo* and *in vivo* models. Integrated with dual targeting to both cancer cells and tumor microenvironment, ZVI nanotherapeutics have profoundly opened up the potential for new advanced cancer therapy with reduced side effects and augmented efficacy.

## Materials and methods

### Preparation of ZVI@Ag and ZVI@CMC NPs

ZVI@Ag and ZVI@CMC NPs were synthesized as described in previous studies 3, 5. In brief, to synthesize ZVI@Ag, ferrous sulfate (FeSO_4_) and trisodium citrate (Na_3_C_6_H_5_O_7_) dehydrate were mixed within deionized water by magnetic stirring. Next, sodium borohydride (NaBH_4_) was added dropwise into the mixture and stirred at room temperature to form ZVI. Finally, silver nitrate (AgNO_3_) was added with stirring to get ZVI@Ag NPs. To synthesize ZVI@CMC, ferrous sulfate (FeSO_4_) and carboxymethyl cellulose (CMC) were mixed within stirred distilled water followed by adding sodium borohydride (NaBH_4_) to the stirred mixture at room temperature for ZVI@CMC NPs assembly. Ultimately, both NPs solutions were washed with ethanol several times and collected using a magnet platform. Preparation of ZVI@Ag or ZVI@CMC NPs was done in an argon gas environment. The hydrodynamic size distribution of ZVI-NPs measured by dynamic light scattering at room temperature was done using Delsa Nano C Particle analyzer (Beckman Coulter, Brea, CA, USA).

### Haemolysis assay

For haemolytic activity analysis, ZVI-NPs were incubated with red blood cells (RBCs) and suspended at 37 °C for 1 h then centrifugated at 3,000 rpm for 5 min. Triton X-100 and PBS were used to generate the positive (100% haemolysis) and the negative (0% haemolysis) controls, respectively. The absorbance of the supernatant was measured at 545 nm.

### Cell lines and culture conditions

Human lung cancer cell lines H1299, H460, A549, mouse Lewis lung carcinoma (LLC), and human lung fibroblast cell lines MRC-5 and IMR-90 were purchased from American Tissue Culture Company (Rockville, MD, USA). Luciferase-LLC (LLC-luc) cell line was provided by Dr. Muh-Hwa Yang (Institute of Clinical Medicine, National Yang-Ming University, Taiwan). These cell lines were maintained in DMEM medium (Gibco, Grand Island, NY, USA). Human monocytic cell line THP-1 was purchased from Bioresource Collection and Research Center (BCRC, Taiwan) and maintained in RPMI 1640 medium (Gibco). Both DMEM and RPMI 1640 media were supplemented with 10% Fetal Bovine Serum (FBS; Gibco) and 1% penicillin/streptomycin (Gibco). Human umbilical vein endothelial cell line (HUVEC) was provided by Dr. Li-Wha Wu (Institute of Molecular Medicine, National Cheng Kung University, Taiwan). HUVEC cells cultured in dishes, which were coated with 0.1% gelatin for 1 h, were maintained with endothelial cell growth medium-2 (EBM-2; Lonza, Walkersville, MD, USA) and supplemented with SingleQuots^TM^ growth factor kit (Lonza). All cells were incubated at 37 °C with 5% CO_2_.

### Mouse splenocytes isolation

Following the sacrifice of C57BL/6 mice, spleens were aseptically harvested and washed three times with PBS. To obtain a single cell suspension, the spleens were crushed and passed through a 70-μm nylon cell strainer (Falcon, Franklin Lakes, NJ, USA), and then red blood cells were lysed and removed. The splenocytes were resuspended thoroughly in RPMI 1640 medium containing 10% FBS and 1% penicillin/streptomycin.

### Regulatory T cells (Tregs) differentiation

Following splenocytes isolation, cells were incubated with RPMI 1640 medium containing 10% FBS, 1% penicillin/streptomycin, plate-bound anti-CD3 and anti-CD28 antibodies (BD Biosciences, San Jose, CA, USA), recombinant mouse 5 ng/mL IL-2 and recombinant human TGF-β for Treg differentiation.

### Luciferase cytotoxic T lymphocyte assay

Following splenocytes isolation, 1 × 10^4^ lymphocytes were then incubated with 1 × 10^3^ LLC-luc cells per well (96-well plate) containing 100 μL RPMI 1640 medium supplemented with 10% FBS and 1% penicillin/streptomycin. During the co-cultivation, naïve lymphocytes were activated by stimulation using plate-bound anti-CD3 and anti-CD28 antibodies, interleukin (IL)-2 (EL-4 culture supernatant) and IL-7 (R&D Systems, Minneapolis, MN, USA), Insulin-Transferrin-selenium (ITS; Gibco), and β-mercaptoethanol (SERVA, Heidelberg, Germany) as previously described 23. After 24 h co-cultivation, culture medium was removed, and cell pellets were rinsed twice with PBS. The number of viable LLC-luc cells was determined by luciferase assays using the Dual-Glo Luciferase Assay System (Promega, Madison, WI, USA) as previously described 24.

### Isolation of mouse bone-marrow-derived macrophages (BMDMs)

Isolated bones were aseptically harvested from hind legs of C57BL/6 mice, and muscle tissues were removed. Bone marrow was flushed out of the bones using a 25-gauge needle attached to a syringe containing BMDM growth medium, which consists of DMEM, 20% L929 cell-conditioned media to generate M-CSF (macrophage colony-stimulating factor), 10% FBS, and 1% penicillin/streptomycin. Then, BMDMs were allowed to differentiate for 7 days at 37 °C with 5% CO_2_, and the growth medium was changed every 2 days during* ex vivo* culture.

### THP-1 macrophage differentiation and polarization

The macrophage-like state was induced by treating THP-1 monocytes for 48 h with 100 ng/mL phorbol 12-myristate 13-acetate (PMA; Sigma-Aldrich, St. Louis, MO, USA) in 6 well-plates at a density of 5 × 10^5^ cells/well. After washing twice with culture medium, the resting macrophages (M0) were treated with 20 ng/mL IFNγ plus 1 μg/mL LPS for 6 h to differentiate into the M1 phenotype or with 20 ng/mL IL-4 for 24 h to the M2 phenotype. Cells were maintained in 5% CO_2_ at 37 °C during differentiation and polarization.

### Co-culture system of macrophages and cancer cells

For LLC/BMDM co-culture system, the lower compartment of a 6-well plate was seeded with BMDMs (1 × 10^6^) while the upper compartment with LLC cells (1 × 10^6^). Cells were cultured with BMDM growth medium containing ZVI-NPs or not for 48 h at 37 °C with 5% CO_2_. BMDMs were collected for further analysis. For A549/THP-1 macrophage co-culture system, the lower compartment of a 6-well plate was seeded with A549 cells (1 × 10^6^) while the upper compartment with THP-1 macrophages (M0) (1 × 10^6^). Cells were cultured with RPMI 1640 medium containing 10% FBS and treated with ZVI-NP or not for 48 h at 37 °C with 5% CO_2_. A549 cells were collected for further analysis.

### Cell viability assay

Cell viability assay was performed to evaluate the cytotoxicity of ZVI-NP by using 3-(4,5-dimethylthiazol-2-yl)-2,5-diphenyltetrazolium bromide (MTT) assay or cell counting kit 8 (CCK-8) assay. After ZVI-NP treatment, cells were replaced with fresh medium containing 1 mg/mL MTT (Sigma-Aldrich) or CCK-8 reagent (Dojindo Laboratories, Kumamoto, Japan) and then incubated at 37 °C for 2 h. For MTT assay, crystals were dissolved in dimethyl sulfoxide, and the optical absorbance at 570 nm was measured. For CCK-8 assay, the optical absorbance at 450 nm was measured.

### Wound healing migration and transwell invasion assays

For wound healing migration assay, A549 and H460 cells (1 × 10^5^) were seeded into each compartment of the culture insert (Ibidi, Martinsried, Germany). A cell-free gap of 500 μm was created after removing the insert, and the cells were treated with ZVI-NPs during the migration process. For transwell invasion assay, 5 × 10^5^ cells were seeded onto the upper side of transwell membrane (Falcon) which was precoated with Matrigel (Corning, New York, NY, USA) one day before seeding the cells. After ZVI-NP treatment for 16 h, the cells attached on the reverse side of the membrane were fixed and stained. Random views of both migration and invasion assay were photographed by Olympus CKX53 (Olympus, Tokyo, Japan) and analyzed by ImageJ software.

### Cancer sphere formation assay

H460 and H1299 cells were seeded in ultra-low attachment 6-well plates with DMEM/F12 containing N-2 supplement (Invitrogen, Foster City, CA, USA), 20 ng/mL epithelial growth factor (PeproTech Inc., Rocky Hill, NJ, USA), 20 ng/mL basic fibroblast growth factor (PeproTech Inc.) and 1% penicillin/streptomycin at 2 × 10^4^ cells per well. Cells were incubated for 7 days and then treated with or without ZVI-NP for 48 h. Cancer spheres consisting of 20 or more cells were photographed and counted.

### Endothelial cell transwell migration assay

HUVEC cells (1 × 10^5^) were seeded into the upper chambers of transwell (Falcon) with serum-free DMEM medium. The lower chambers were filled with DMEM medium containing 20% FBS plus ZVI-NPs and then incubated at 37 °C for 24 h. The cells attached on the reverse side of the membrane were stained with crystal violet and counted under an upright microscope (Nikon E400, Tokyo, Japan).

### Tube formation assay

Phenol Red-free Matrigel (Corning) was added to 96-well plates and then incubated at 37 °C for 1 h. HUVEC cells (2 × 10^4^ per well) were seeded into 96-well plates with culture medium containing ZVI-NPs or not and then incubated for 8 h. Tube formation was observed and photographed randomly under microscope (Nikon E400).

### Intracellular ROS and lipid peroxidation measurement

To measure intracellular ROS level, cells (8 × 10^4^ per well) were seeded in 12-well plates. After treatment with ZVI-NP, cells were detached with trypsin, washed twice with PBS, and then incubated with 10 μM H_2_DCFDA (Sigma-Aldrich) at 37 °C for 30 min in the dark. After washing with PBS, the intracellular ROS levels were analyzed by flow cytometry (CytoFLEX^TM^, Beckman coulter, Brea, CA, USA). To detect lipid peroxides level, cells were incubated with 5 μM Liperfluo (Dojindo) at 37 °C for 30 min in the dark. To measure intracellular 4-HNE level, cells were incubated with the primary antibody for 30 min on ice followed by Alexa Fluor 488 tagged secondary antibody for 30 min. Sample fluorescence was measured by flow cytometry (CytoFLEX^TM^).

### Mitochondrial ROS and mitochondrial membrane potential assay

To measure mitochondrial ROS, cells were incubated with 5 μM MitoSOX (Invitrogen) at 37 °C for 30 min in the dark. Sample fluorescence was measured by flow cytometry (CytoFLEX^TM^). To detect mitochondrial membrane potential, cells were incubated with 20 nM 3,3-Dihexyloxacarbocyanine iodide (DiOC6) (Enzo, New York, NY, USA) for 15 min or 5 μg/mL Rhodamine 123 (Sigma-Aldrich) for 30 min at 37 °C in the dark and then analyzed by flow cytometry (CytoFLEX^TM^).

### Mitochondrial respiration function measurement

Cell monolayers were cultured in XF Cell Culture Microplates (Seahorse Bioscience, North Billerica, MA, USA) at a density of 2.5 × 10^4^ cells per well. The sensor cartridge (Seahorse Bioscience) was polarized overnight and calibrated. After ZVI-NP treatment, the medium was replaced with appropriate assay medium without sodium bicarbonate and serum, and cells were then incubated for 30 min at 37 °C without CO_2_. The compounds were injected sequentially: 1 μM oligomycin; 2 μM FCCP; 2 μM Rotenone (all from Sigma-Aldrich). The basal OCR and OCR responses toward compounds injection were performed in a Seahorse XF24 analyzer (Seahorse Bioscience) according to the manufacturer's instructions.

### Total ATP and NADPH determination assay

Total ATP level was measured by ATP determination kit (Invitrogen). Cells (8 × 10^4^ per well) were seeded in 12-well plates. After ZVI-NP treatment, cells were lysed and mixed with 1X reaction solution, and then incubated for 5 to 15 min. Then, the sample analysis was performed according to the manufacturer's instructions.

To detect NADPH level, NADPH determination assay kit (Biovision, San Francisco, CA, USA) was employed. Cells (8 × 10^5^) were seeded in 10 cm dish. After ZVI-NP treatment, cells were lysed by extraction buffer and then heated 60 °C for 30 min. The extracted samples were then applied to each well of a 96-well plate and mixed with NADPH developer at room temperature for 1 to 4 h incubation. To determine the intracellular NADPH level, the optical absorbance at 450 nm was measured once an hour according to the manufacturer's instructions.

### Transfection of plasmids

NRF2 plasmid (pCMV3-C-OFP/NFE2L2) was purchased from Sino Biological Inc. (Beijing, China), and transfection was conducted using Turbofect reagent (Invitrogen) according to the manufacture's protocol. After 24 h transfection, cells were collected for the analysis of lipid peroxidation or harvested for animal experiments.

### RNA extraction and RT-qPCR assays

After ZVI-NP treatment, total RNA was extracted using Trizol reagent (Invitrogen). Purified RNA was converted into cDNA by reverse transcription. RT-qPCR was performed with SYBR Green Master Mix (Invitrogen) using the StepOnePlus™ Real-Time PCR system (Life Technologies, Carlsbad, CA, USA). Expression levels were normalized with internal control β-actin. The primer sets are listed in Table S1.

### Protein extraction and Western blotting

Cells were lysed in 1× RIPA buffer (0.05 M Tris-HCl, 0.15 M sodium chloride, 0.25% deoxycholic acid, 1% Nonidet P-40, 1 mM EDTA, 0.5 mM DTT, 1 mM phenylmethylsulfonyl fluoride, 5 μg/mL leupeptin, and 10 μg/mL aprotinin) containing protease inhibitors cocktail (Sigma-Aldrich). Lysates were centrifuged at 13,200 rpm for 15 min. Protein extracts were solubilized in loading buffer (60 mM Tris-base, 2% SDS, 10% glycerol, and 5% β-mercaptoethanol). Equal amounts of lysate were separated on 8% SDS-PAGE and transferred onto a polyvinyl difluoride (PVDF) membrane. The protein was identified by incubating the membrane with primary antibodies followed by horseradish peroxidase-conjugated secondary antibodies. The antibody conditions are described in Table S2.

### Chromatin immunoprecipitation assay (ChIP assay)

Cells (5 × 10^6^) were cross-linked followed by preparation of nuclear lysates using Magna ChIP^TM^ protein G Kit (Millipore, Burlington, MA, USA). Nuclear lysates were sonicated to shear DNA to around 500 bp followed by immunoprecipitation for 16 h at 4 °C using IgG or anti-NRF2 antibody (Genetex, San Antonio, TX, USA). The levels of targeted genes in ChIP products were determined by RT-qPCR. Primers used are listed in Table S1.

### Immunofluorescence (IF) and immunochemistry (IHC) assay

For immunofluorescence (IF) staining, Opal stain kit (PerkinElmer, Waltham, MA, USA) was employed. After cell fixation, antigen retrieval was performed with citrate buffer (pH 6.0) in a microwave oven. The slides were then washed, blocked, and incubated with β-TrCP primary antibody at 4 °C overnight followed by incubation with secondary antibody polymer HRP for 10 min and subsequently with Opal fluorophore for 10 min at room temperature. To further stain with anti-NRF2 antibody, the slides were again placed in citrate buffer (pH 6.0) and heated in a microwave oven. After Opal staining process, DAPI was applied for nuclei staining.

For immunochemistry (IHC) staining, Novolink Max Polymer kit (Leica Biosystems, Wetzlar, Germany) was employed. All slides were dewaxed with xylene/ethanol, and then antigen retrieval was performed with TRS buffer (pH 6.0) in a microwave oven. After blocking, the slides were reacted with primary antibodies. The peroxidase activity was visualized with diaminobenzidine tetrahydroxychloride (DAB) solution. The sections were counter stained with hematoxylin. Dark brown staining was considered positive. The antibody conditions are described in Table S2.

### Transmission electron microscopy (TEM) imaging

The ZVI-NPs were characterized by TEM as previously described 3, 5. For intracellular structure observation, cells were collected and incubated overnight with fix solution (2.5% glutaraldehyde, 3 mM CaCl_2_, and 0.1 M cacodylate). Each sample was diluted with absolute alcohol and then applied onto copper grids followed by vacuum drying. The digital images were acquired using a JEOL JEM1400 TEM (JEOL, Tokyo, Japan).

### Animal study - immunodeficient and immunocompetent models

All animal experiments were performed in compliance with institutional guidelines for use and care of animals. For H460 xenograft model of immunodeficient mouse, 5-6-week-old BALB/c nude mice (ZVI@Ag treatment) or NOD/SCID mice (ZVI@CMC treatment) were subcutaneously implanted with 1 × 10^6^ H460 cells. For A549 xenograft model of immunodeficient mouse and spontaneous lung metastasis model, 5-6-week-old NOD/SCID mice were subcutaneously implanted with 5 x 10^6^ A549 cells. For experimental lung metastasis model, H460 cells (1 × 10^6^ cells/200 μL) were resuspended in serum-free medium and injected intravenously (i.v.) into tail-vein of NOD/SCID mice. For subcutaneous model of immunocompetent mouse, LLC cells (5 × 10^5^) were injected into both flank of 6-week-old C57BL/6 mice.

When tumor volume reached 40-50 mm^3^, the mice were i.v. injecteded with PBS or ZVI-NPs at the doses and times indicated. Mice were weighed, and the volumes of the xenografts or allografts were measured and quantified during expreiment. The tumor volume was calculated as (length × width square)/2 in mm^3^. For the tumor metastasis assay, lung was excised and fixed with 4% formaldehyde (Sigma-Aldrich) at the end of expreiemt. Area of metastatic lung tumor nodules was analyzed by imageJ.

### Animal study - humanized mice animal model* in vivo*

To generate humanized mice, human peripheral blood mononuclear cells (hPBMCs) were i.v. injected into 6-8-week-old advanced severe immunodeficiency (ASID) mice (NOD.Cg-Prkdc^scid^Il2rg^tm1Wjl^/ YckNarl; National Laboratory Animal Center, Taipei, Taiwan). Eight days after hPBMC engraftment, H460 cells (5 × 10^6^) were subcutaneously injected into the right flank of hPBMC-ASID mice. When tumor xenograft volume reached 50-200 mm^3^, the mice were treated i.v. with 25 mg/kg ZVI@CMC four times every other day. Mice were weighed, and the xenografts were measured and quantified as described above.

### Single cell suspension of tissue and flow cytometry analysis

Tumor tissues were digested with 0.1 mg/mL collagenase (Sigma-Aldrich) and 1 mg/mL dispase II (Sigma-Aldrich) in serum-free DMEM for 30 min at 37 °C, and then crushed through mesh for single cell suspension. To determine macrophage polarization, cells from BMDMs, tumor tissues or peripheral blood mononuclear cells were stained with anti-mouse CD11b, CD86, and CD206. To measure the proportion of Tregs, cells from splenocytes, tumor tissues or peripheral blood mononuclear cells were stained with anti-mouse CD25, Foxp3, and CD4. To determine the presentation of PD-1 and CTLA4 on CD8^+^ lymphocytes, cells from splenocytes, tumor tissues or peripheral blood mononuclear cells were stained with anti-mouse PD-1, CTLA4, and CD8. For humanized hPBMC-ASID mice, cells from tumor tissues or peripheral blood mononuclear cells were stained with anti-human CD45, CD3, CD4, CD8, and CD25. The antibodies conditions are described in Table S2. After staining process by following the manufacturer's instructions, samples were analyzed by flow cytometry (CytoFLEX^TM^).

### Organ distribution of iron

BALB/c nude mice (5-6-week-old) were i.v. injected with 25 mg/kg ZVI-NPs or PBS. After treatment, major organs (heart, lungs, spleen, liver, and kidney), tumors and blood were collected at the indicated time. Each organ was homogenized and dissolved in nitrohydrochloric acid. The sample solutions were continuously shaken for 2 days to ensure iron dissociation. All samples were filtered and analyzed using an inductively coupled plasma mass spectrometry (Agilent technology, Santa Clara, CA, USA) provided by Chia Nan University of Pharmacy and Science, Taiwan.

### Statistical analysis

Three independent experiments for cell studies and six mice per group for animal studies were analyzed unless indicated otherwise. Two-tailed Student's t-test was used in cell and animal studies. Data represent mean ± s.e.m. The levels of statistical significance were expressed as *p*-values, *, *p* < 0.05; **, *p* < 0.01; ***, *p* < 0.001; ns: non-significant.

## Results

### ZVI-NP exhibits cancer-specific cytotoxicity

**Figure 1:**
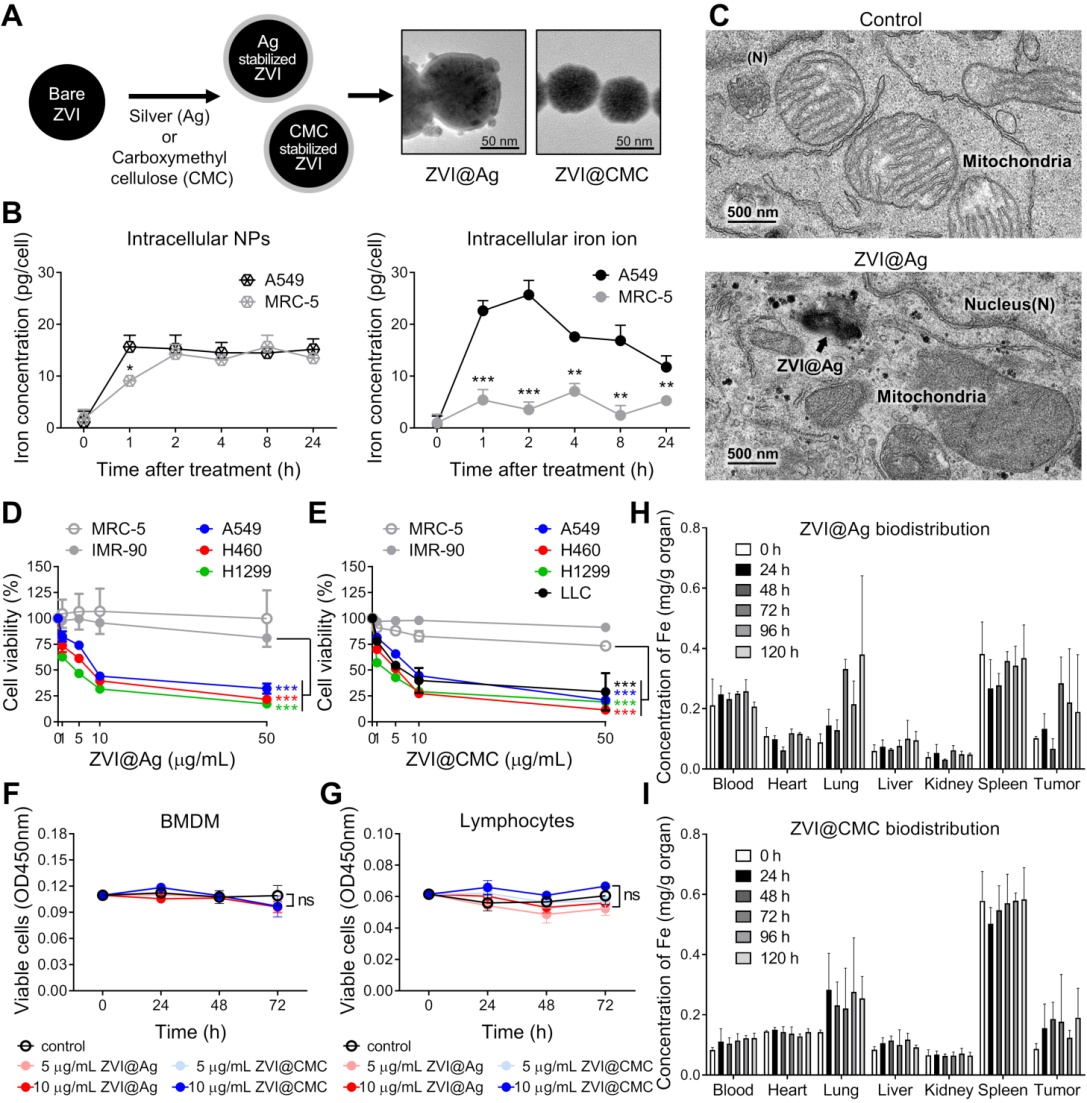
Morphological and biological characterization of ZVI-NPs in cancer-specific cytotoxicity.** A,** TEM images showing the round shape morphology and surface coating of both types of NPs. Scale bar: 50 nm. **B,** Intracellular concentrations of ZVI@CMC NPs (*left*) and iron ions (*right*) were determined in A549 and MRC-5 cells after treatment with ZVI@CMC NPs (10 mg/mL) for 1, 2, 4, 8, or 24 h. **C,** TEM images showing the presence of ZVI@Ag (arrow) and the mitochondria with damaged cristae 24 h after NPs (10 μg/mL) treatment. **D** and** E,** MTT assay showing both ZVI@Ag NPs (D) and ZVI@CMC NPs (E) dose-dependently inhibited cell viability in lung cancer cells H460, A549 and H1299 without affecting lung fibroblast cells MRC-5 and IMR-90 after 48 h of treatment. **F** and **G,** Both ZVI@Ag NPs or ZVI@CMC NPs did not inhibit cell viability of *ex vivo* isolated BMDMs (F) and splenic lymphocytes (G) in the test concentrations (5 and 10 μg/mL) in the 72 h of treatment period. **H** and **I,** Biodistribution of ZVI@Ag NPs (H) or ZVI@CMC NPs (I) in major organs in nude mice given i.v. injection of a single dose (50 mg/kg). The iron concentrations in tissue samples were quantified at indicated time (*n* = 5 per group). Data were mean ± s.e.m. (*n* = 3 for cell-based assays). ns: non-significant; *, *p* < 0.05; **, *p* < 0.01; ***, *p* < 0.001.

We have developed two types of ZVI-NP, including silver coated (ZVI@Ag) and carboxymethylcellulose coated (ZVI@CMC) NPs. In this study, we evaluated the anti-cancer efficacy and immune modulation potential of our newly developed ZVI-NP in lung cancer models using various *in vitro, in vivo* and *ex vivo* models as illustrated in the schematic in Figure S1A. As shown in the TEM images of** Figure 1A** and Figure S1, both types of NPs exhibited core/shell structure and the mean physical diameters of ZVI@Ag NPs and ZVI@CMC NPs were 81.08 ± 14.29 nm and 70.17 ± 14.4 nm, respectively (Figure S1C, F). On the other hand, the dynamic light scattering analysis revealed that both the freshly prepared ZVI@Ag and ZVI@CMC NPs were monodispersed, and the mean hydrodynamic sizes were 97.1 ± 19.3 nm and 78.8 ± 19.8 nm, respectively (Figure S1D, G). The elemental composition of ZVI@Ag has been determined in our previous work 3, and we further performed FTIR analysis to confirm the successful coating of CMC on ZVI@CMC (Figure S1H). Additionally, zeta potentials of ZVI@Ag and ZVI@CMC NPs were measured and shown in Figure S1I-J. Of note, as shown in Figure S1K-L, both ZVI@Ag and ZVI@CMC NPs have no significant haemolysis effects according to ISO10993-4.

We further evaluated the uptake of ZVI@CMC in human lung cancer cells (A549) versus nonmalignant human lung fibroblast cells (MRC-5) by measuring the intracellular iron ion levels compared to that of the untreated control group (**Figure 1B**). Although the concentrations of ZVI@CMC NPs were detected in both lung cancer and normal lung cells at the early time points (1 h and 2 h) (*left*, **Figure 1B**), the intracellular iron ions increased rapidly in A549 cells, but not in MRC-5 cells (*right*, **Figure 1B**). Similar results were observed for ZVI@Ag NPs where the conversion of ZVI to intracellular iron ions has been reported to be processed rapidly *via* acidification of endosome-lysosome system in oral squamous cell carcinoma lines than in normal cells 3, suggesting a cancer cell specific enhanced conversion of ZVI-NP.

We next examined the ultrastructure of ZVI-NP inside cancer cells. The TEM images illustrated the presence of ZVI@Ag NPs in lysosomes (*arrow*,** Figure 1C**) and the release of iron particles into cytoplasm in NPs-treated A549 cancer cells. Notably, these cells exhibited ruptured mitochondrial outer membrane and reduced mitochondrial crista with normal nuclear size and lack of chromatin condensation (*lower*,** Figure 1C**). These organelle alterations resembled the morphological characteristics of the novel iron-dependent programmed cell death, namely ferroptosis, as described previously 6, 7, 25.

To evaluate the cytotoxic effects induced by ZVI@Ag or ZVI@CMC NPs, human non-small cell lung cancer cell lines (H460, A549, and H1299), murine Lewis lung carcinoma cell line (LLC), and non-malignant human lung fibroblast cell lines (MRC-5 and IMR-90) were treated with various doses of ZVI@Ag or ZVI@CMC NPs for 48 h and then subjected to MTT cell viability assay (**Figure 1D-E**). Both ZVI@Ag and ZVI@CMC NPs treatments significantly inhibited the viability of lung cancer cells without showing apparent cytotoxicity toward non-malignant lung cell lines. Given the observation of cancer-specific cytotoxicity, we further examined the viability of additional normal cells including *ex vivo* isolated bone-marrow-derived macrophages (BMDMs) and splenic lymphocytes from C57BL/6 mice after *ex vivo* treatment of ZVI@Ag or ZVI@CMC NPs (**Figure 1F-G**). Neither treatment of the ZVI-NPs affected the viability of *ex vivo* cultured BMDMs or lymphocytes, supporting that ZVI-NP exerted cancer-specific cytotoxicity.

Certain amount of iron is essential for organismal functions. However, excessive amounts of iron are associated with ROS generation and cytotoxicity responses 26, 27. To dissect the biodistribution of iron from the NPs *in vivo*, we measured the concentrations of iron in organs of nude mice bearing subcutaneous A549 xenografts treated with a single i.v. injection of ZVI-NPs (25 mg/kg) (**Figure 1H-I**). The results indicated that both ZVI@Ag and ZVI@CMC NPs retained in the tumor and lung for 120 h after single dose of i.v. injection. Notably, iron signal was relatively high in spleen tissue in the control group due to rich in hemoglobin background. Collectively, these findings revealed that ZVI-NP treatment exerted cancer-specific cytotoxicity in lung cancer models *in vitro*, and ZVI-NPs were preferentially retained in the tumor lesions and lung tissue *in vivo*.

### ZVI-NP suppresses cancer stemness and angiogenesis

**Figure 2:**
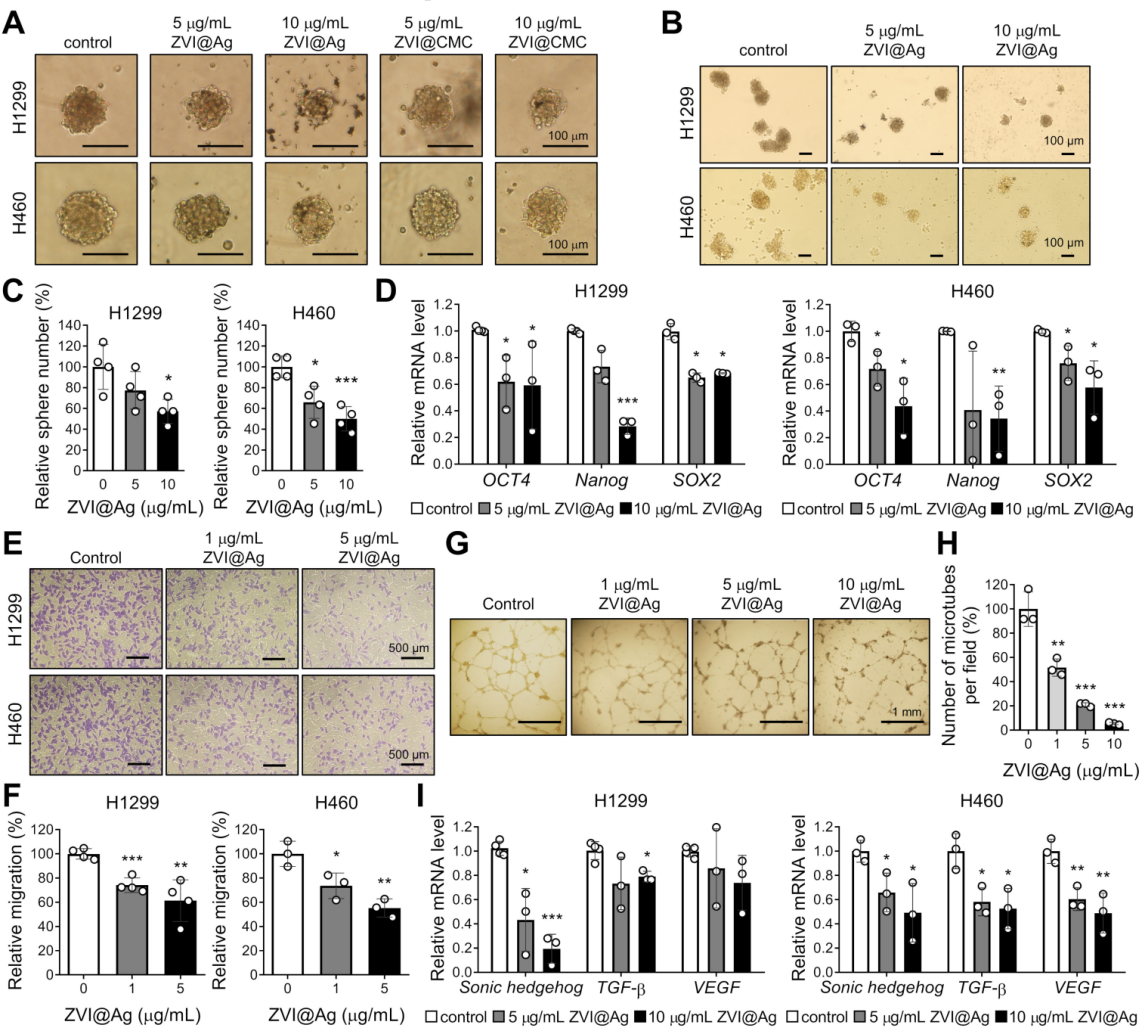
ZVI-NP showed anti-cancer stemness and anti-angiogenesis effects *in vitro*.** A-C,** Representative images of the size (A) and the number (B) as well as the quantitative analysis of cancer spheres (C) derived from H1299 and H460 cells treated with ZVI-NPs were measured under an inverted light microscope. **D,** Expression of cancer stemness genes was measured by RT-qPCR after ZVI@Ag NPs treatment for 48 h in H1299 (*left*) and H460 (*right*). **E** and **F,** Representative transwell migration images (E) and its quantitative analysis (F) of HUVEC endothelial cells cultured in CM derived from H1299 or H460 cells treated with or without ZVI@Ag NPs for 8 h. **G** and **H,** Representative tube formation microscopy images (G) and its quantitation (H) of HUVECs treated with ZVI@Ag NPs for 16 h. **I,** Expression of pro-angiogenesis genes was measured by RT-qPCR after ZVI@Ag NPs treatment for 48 h in H1299 (*left*) and H460 (*right*). The expression levels of mRNA were normalized to *β-actin*. Data were mean ± s.e.m. (*n* = 3). *, *p* < 0.05; **, *p* < 0.01; ***, *p* < 0.001.

In addition to the observed cancer-specific cytotoxicity induced by ZVI-NP, we further evaluated the inhibitory effects of ZVI-NP on cancer stemness and angiogenesis in H460 and H1299 cells. As shown in **Figure 2A**, both ZVI@Ag and ZVI@CMC NPs decreased the size of tumor sphere as compared to the control. The number of spheres was significantly reduced by ZVI@Ag NPs treatment (**Figure 2B-C**). Importantly, RT-qPCR analysis showed that ZVI@Ag NPs treatment significantly decreased the expression levels of cancer stemness genes, including *OCT4*, *Nanog*, and *SOX2* (**Figure 2D**). Moreover, ZVI-NP treatment significantly suppressed both the migration and invasion abilities of cancer cells (Figure S2A-C). Together, these findings showed that ZVI-NP treatment exerted anti-cancer stemness effects on lung cancer cells.

Further, we discovered that conditioned medium (CM) derived from ZVI@Ag NPs-treated cancer cells for 8 h significantly decreased the migration ability of human umbilical vein endothelial cells (HUVECs) (**Figure 2E-F**) and the number of tube formation (**Figure 2G-H**) without apparent reduced viability of HUVECs (Figure S2D). In addition, the expressions of pro-angiogenesis genes such as *Sonic hedgehog*, *TGF-β* and *VEGF* were downregulated after ZVI@Ag NPs treatment (**Figure 2I**). Collectively, these results indicated that ZVI-NP treatment inhibited both cancer stemness and angiogenesis *in vitro*, which may contribute to their integrated anti-cancer efficacy.

### ZVI-NP causes lipid peroxidation and ferroptosis in cancer

**Figure 3:**
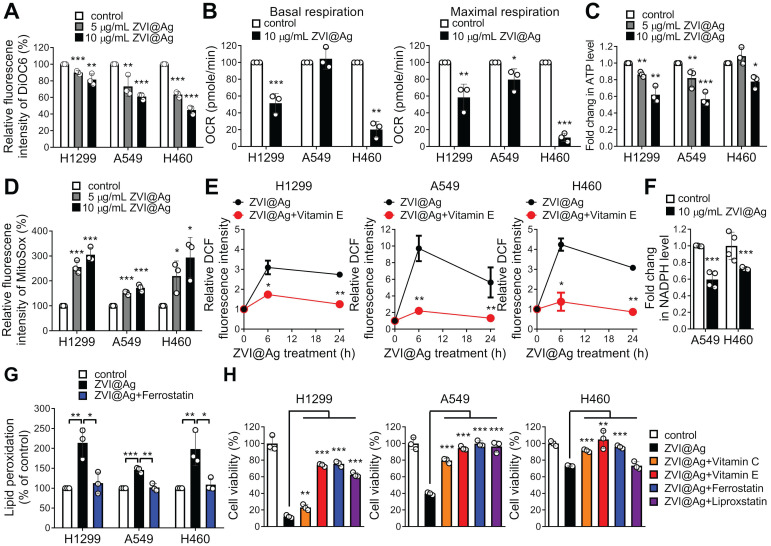
ZVI-NP caused mitochondria dysfunction, oxidative stress, and lipid peroxidation *in vitro*.** A,** Mitochondrial membrane potential was analyzed by flow cytometry analysis of DiOC6 after ZVI@Ag NPs treatment. **B,** Oxygen consumption rate (OCR) in terms of basal (*left*) or maximum (*right*) respiration was examined using seahorse XF24 analyzer after ZVI@Ag NPs (10 μg/mL) treatment for 24 h. **C,** Bioluminescence assay showed that total ATP levels were dose-dependently suppressed in all three cancer lines by ZVI@Ag NPs treatment for 24 h. **D,** Mitochondrial ROS in the three cancer lines was analyzed by flow cytometry after ZVI@Ag NPs treatment. **E,** Intracellular ROS levels were measured by flow cytometry after ZVI@Ag NPs (5 μg/mL) treatment with or without Vitamin E (100 μM). **F,** Colorimetric analysis was performed to detect NADPH levels after ZVI@Ag treatment for 24 h. **G,** Flow cytometry analysis of lipid peroxidation for the three cancer cells treated with ZVI@Ag NPs (5 μg/mL) with or without Ferrostatin (10 μM) pre-treatment. **H,** Cell viability was determined after co-treatment with ZVI@Ag NPs (10 μg/mL) and Vitamin C (100 μM), Vitamin E (100 μM), Ferrostatin (10 μM), or Liproxstatin (10 μM) for 48 h. Data were mean ± s.e.m. (*n* = 3). *, *p* < 0.05; **, *p* < 0.01; ***, *p* < 0.001.

Given the observation of morphological evidence of ferroptosis shown in **Figure 1C**, we further determined whether the function of mitochondria was affected by ZVI-NP treatment. As shown in **Figure 3A** and Figure S3A, ZVI-NPs treatment significantly decreased the fluorescence intensities of DiOC6 and Rhodamine 123, indicating the loss of mitochondrial membrane potential in treated cancer cells H1299, H460 and A549. Subsequently, seahorse assay and ATP production analysis demonstrated reduced oxygen consumption rate (OCR) (**Figure 3B**) and ATP level (**Figure 3C**) after ZVI@Ag NPs treatment. Furthermore, the MitoSOX fluorescence intensity was significantly augmented in ZVI@Ag NPs-treated cells, indicating the accumulation of mitochondrial reactive oxygen species (mtROS) and increased oxidative stress in mitochondria (**Figure 3D**). Together, these findings confirmed that ZVI@Ag NPs could induce mitochondria dysfunction and overproduction of mtROS in lung cancer cells.

Since damaged mitochondria could release high levels of ROS into the cytoplasm, and intracellular ZVI may also generate ROS through the Fenton reaction, we next determined the intracellular ROS level in cancer cells after treatment. As shown in **Figure 3E**, ZVI@Ag NPs treatment drastically increased the intensity of DCF fluorescence, an index of intracellular ROS. Similar result was found after treatment with ZVI@CMC NPs (Figure S3B). In addition, the intracellular NADPH level, indicating the ROS detoxification power, was also declined in cancer cells after ZVI@Ag treatment (**Figure 3F**), supporting the notion that ZVI-NP treatment diminished antioxidant defense systems and induced intracellular ROS and mtROS levels to augment oxidative stress.

To confirm whether iron overload and oxidative stress triggered peroxidation of membrane lipid, flow cytometry analysis of lipid peroxidation status was conducted (**Figure 3G**; Figure S3C). The level of lipid peroxidation was significantly increased after ZVI-NP treatment, and this increment was inhibited by the addition of canonical ferroptosis inhibitor, Ferrostatin-1, in all lung cancer cells examined. These results demonstrated that ZVI-NP treatment caused excessive production of ROS and led to ferroptotic lipid peroxidation in cancer cells.

To further verify whether ZVI-NP treatment-induced cancer-specific cell death was attributed to high levels of ROS and ferroptotic lipid peroxidation, ZVI-NP-treated cancer cells were incubated with antioxidant vitamins (vitamin C or vitamin E), ferroptosis inhibitor (Ferrostatin-1) or lipid peroxidation inhibitor (Liproxstatin-1) and then subjected to ROS measurement or cell viability assay. Notably, ZVI-NP-induced intracellular ROS level was significantly suppressed by the addition of antioxidant vitamin E (**Figure 3E**; Figure S3B). Moreover, ZVI-NP-induced cell death was attenuated by vitamin C, vitamin E, Ferrostatin-1 or Liproxstatin-1 treatments (**Figure 3H**; Figure S3D), indicating that the cancer-specific cytotoxicity induced by ZVI-NP depends on excessive oxidative stress and could be mainly attributed to ferroptosis. Collectively, we identified a ferroptotic cancer cell death model by which ZVI-NP induced excessive ROS and resulted in peroxidation of membrane lipid, thereby causing ferroptosis of lung cancer cells.

### ZVI-NP suppresses NRF2-mediated cytoprotection program

**Figure 4:**
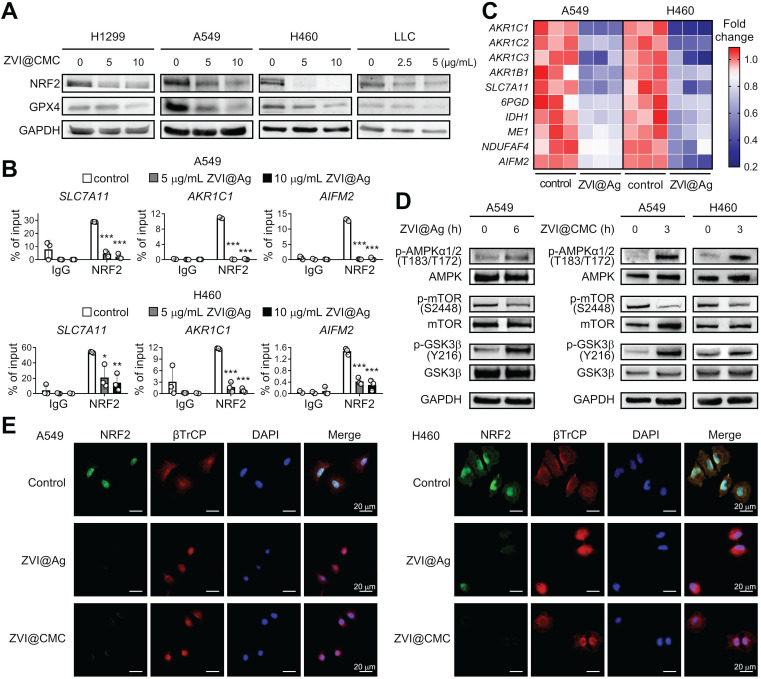
ZVI-NP inhibited NRF2-regulated antioxidant activity *via* enhancement of GSK3β/β-TrCP degradation pathway.** A,** Immunoblotting of NRF2 and GPX4 in four cancer cell lines treated with ZVI@CMC NPs at the indicated doses for 24 h. GAPDH was used as internal control. **B,** ChIP-qPCR assay was performed to measure NRF2 binding ability to the promoter region of *SLC7A11*, *AKR1C1* and *AIFM2* in cells treated with ZVI@Ag NPs in A549 (*upper*) and H460 (*lower*). **C,** mRNA expression of NRF2 downstream genes was measured by RT-qPCR after ZVI@Ag NPs treatment in A549 and H460 cells. The heat maps reflect downregulation of the mRNA levels of these genes compared to the respective untreated controls. **D,** Immunoblotting of p-AMPK, total AMPK, p-mTOR, total mTOR, p-GSK3β and total GSK3β in cells treated with ZVI@Ag NPs (*left*) or ZVI@CMC NPs (*right*) for the indicated time. **E,** Immunofluorescence staining of NRF2, β-TrCP and DAPI in A549 (*left*) and H460 (*right*) cells after ZVI@Ag NPs or ZVI@CMC NPs treatment. Data were mean ± s.e.m. (*n* = 3). *, *p* < 0.05; **, *p* < 0.01; ***, *p* < 0.001.

We further dissected the molecular mechanism underlying the anti-cancer efficacy through ferroptosis induction by ZVI-NP treatment. NRF2, an essential transcription factor, plays an important role in the maintenance of the cellular redox status *via* regulating detoxification, antioxidant, and NADPH regeneration enzymes 28. Interestingly, we showed that protein expression levels of NRF2 and glutathione peroxidase 4 (GPX4), a major scavenger of phospholipid peroxides, were both reduced in lung cancer cells after ZVI-NP treatment (**Figure 4A**; Figure S4A). Furthermore, the binding activities of NRF2 to the targeting promoter region of antioxidant genes *SLC7A11* and *AKR1C1* and the novel anti-ferroptotic gene *AIFM2* 29, 30 were significantly attenuated upon ZVI@Ag treatment in H460 and A549 cells as suggested by chromatin immunoprecipitation (ChIP)-qPCR assay (**Figure 4B**). It is worth to note that the expression levels of NRF2 targeting antioxidant gene *SLC7A11* and ROS detoxification genes *AKR1B1*, *AKR1C1*, *AKR1C2* and *AKR1C3* were decreased after ZVI-NP treatment (**Figure 4C**; Figure S4B). In addition, ZVI@Ag NPs attenuated the expression of genes coding for NADPH-production enzymes such as *IDH1, ME1* and *6PGD* and NADPH-dependent enzymes *NDUFAF4* and *AIFM2* (**Figure 4C**), consistent with our observation of reduced intracellular NADPH levels (**Figure 3F**). Together, these findings revealed a disruption of NRF2-dependent transcription program by which ZVI-NP decreased NRF2 protein level and suppressed its transcription activity, and subsequently decreased the expression of NRF2-regulated ROS detoxification genes.

To further verify whether ZVI-NP-induced inhibition of NRF2 pathway was the major cause of the ferroptosis, we overexpressed NRF2 in lung cancer cells and determined the level of 4-HNE, a biomarker of lipid peroxidation, upon ZVI-NP treatment. As shown in Figure S4C, the intracellular level of 4-HNE was increased after ZVI-NPs treatment. Of note, this increment was largely diminished by NRF2 overexpression, suggesting that ZVI-NP-induced ferroptotic lipid peroxidation was predominantly caused by the inhibition of NRF2. Collectively, our results indicated that the modulation of NRF2 pathway by ZNI-NPs induced ROS generation, peroxidation of membrane lipid and ferropototic cell death.

### ZVI-NP enhances GSK3β/β-TrCP-dependent degradation of NRF2

NRF2 protein level is regulated through degradation pathways. The major pathway is localized in the cytoplasm and governed by KEAP1 E3 ubiquitin ligase 31. The second pathway is in the nucleus and is regulated by GSK3β/β-TrCP phosphorylation-dependent ubiquitination system 32. The KEAP1-dependent degradation of NRF2 is deficient in A549 and H460 cells 33, 34. Thus, we hypothesized that GSK3β/β-TrCP-dependent degradation system may be activated by ZVI-NP treatment. As shown in **Figure 4D**, ZVI-NP treatment induced GSK3β phosphorylation on Tyr216, which is positively correlated with GSK3β activity. Further, immunofluorescence staining illustrated that β-TrCP translocated into the nucleus along with reduced NRF2 protein expression after ZVI-NP treatment (**Figure 4E**). These findings indicated that both ZVI@Ag and ZVI@CMC NPs could enhance NRF2 degradation through the GSK3β/β-TrCP pathway.

To further dissect the upstream signaling that triggered phosphorylation and activation of GSK3β, we examined the level of mammalian target of rapamycin (mTOR), a critical regulator of cell growth and metabolic reprogramming through suppression of GSK3-mediated substrate phosphorylation 35. Interestingly, the immunoblotting results showed that ZVI-NP treatment decreased mTOR phosphorylation on Ser2448 (**Figure 4D**), indicating an antagonistic relationship between mTOR and GSK3β in ZVI-NP-treated A549 and H460 cells. Conversely, the phosphorylation level of AMP-activated protein kinase (AMPK), a pivotal cellular energy sensor that negatively regulates the mTOR pathway, was increased after ZVI-NP treatment (**Figure 4D**). Collectively, these findings identified a novel phosphorylation-dependent NRF2 protein degradation mechanism that ZVI-NP may disrupt AMPK/mTOR pathway to activate p-GSK3/β-TrCP and in turn degrade NRF2, leading to cell death under oxidative stress and subsequent lipid peroxidation.

### ZVI-NP inhibits NRF2 activity and lung metastases* in vivo*

**Figure 5:**
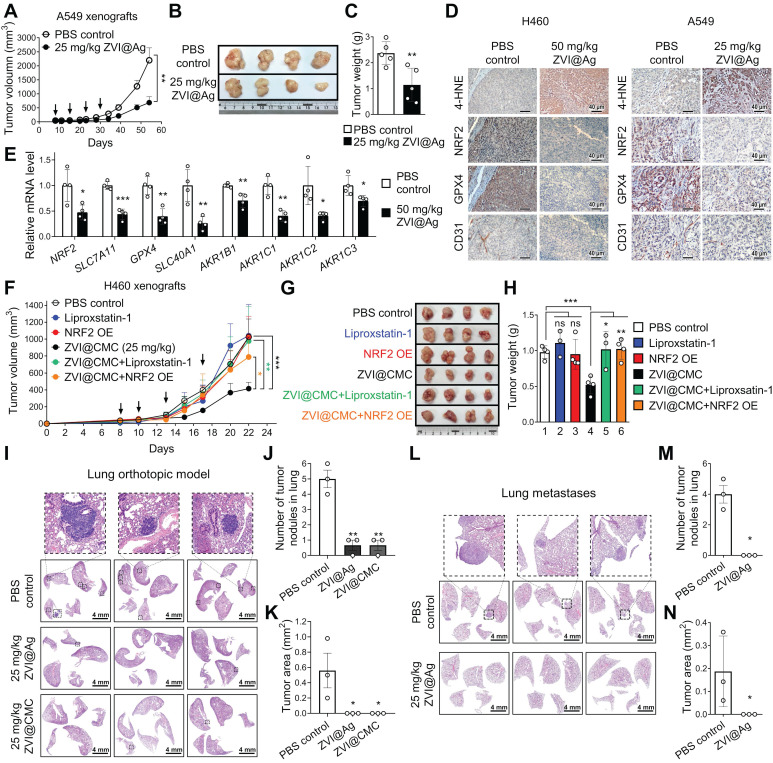
ZVI-NP inhibited NRF2-regulated antioxidant transcription program *in vivo* and suppressed lung metastases.** A-C,** The tumor volume (A), the representative tumor images of the last time point (B), and the final tumor weight (C) of NOD/SCID mice bearing A549 xenografts treated with ZVI@Ag NPs or PBS by i.v. injection once a week (indicated by arrows) (*n* = 5 for each group). **D,** Immunohistochemistry revealed the expression of 4-HNE, NRF2, GPX4 and endothelial cells marker CD31 in tumor tissues from H460 xenografts (*left*) and A549 xenografts (*right*) with or without ZVI@Ag NPs treatment. **E,** Downregulation of NRF2 targeting genes in H460 xenografts treated with 50 mg/kg ZVI@Ag NPs was determined by RT-qPCR.** F-H,** The tumor volume (F), the representative tumor images at the endpoint of study (G), and the respective tumor weight (H) of NOD/SCID mice bearing H460 xenografts or NRF2-overexpressing H460 xenografts treated with ZVI@CMC NPs or PBS by 4 episodes of i.v. injection (indicated by arrows). Liproxstatin-1 (10 mg/kg) treatment was conducted by daily i.p. injection for 10 days. **I-K,** Histopathology of the lung tissues (I) dissected on day 21 from orthotopic mice that received tail vein injection of H460 cells then subjected to i.v. injection of ZVI-NPs or PBS every three days for five times. Quantification of the tumor nodule number (J) and nodule area (K). **L-N,** Histopathology of the lung tissues (L) dissected on day 54 from mice subcutaneously implanted with A549 cells then subjected to i.v. injection of ZVI@Ag NPs or PBS once a week for four weeks. Quantification of the tumor nodule number (M) and nodule area (N). Data were mean ± s.e.m. *, *p* < 0.05; **, *p* < 0.01; ***, *p* < 0.001.

To determine whether ZVI-NP treatment could induce ferroptosis and suppress NRF2-mediated transcriptional regulation of antioxidant functions *in vivo*, subcutaneous xenograft animal models were established. As shown in **Figure 5A-C**, tumor volume, tumor image and tumor weight of A549 xenografts were significantly reduced after i.v. injection of ZVI@Ag NPs as compared to the PBS control. Similar results were obtained in H460 xenograft model treated through intraperitoneal (i.p.) injection of ZVI@Ag NPs (Figure S5A-C). Body weight, blood biochemistry analysis and H&E staining of tissue sections from major organs showed no apparent pathological effects of ZVI@Ag NPs treatment *via* either i.v. or i.p. route (Figure S5D-I).

The IHC staining of xenograft tumor tissues showed that 4-HNE, an index of lipid peroxidation, was dramatically increased after ZVI-NP treatment (**Figure 5D**; Figure S5J). Conversely, protein levels of NRF2 and GPX4 of xenograft tumor tissues were significantly reduced by ZVI-NP exposure (**Figure 5D**; Figure S5J). Interestingly, CD31 staining in ZVI@Ag NPs-treated group showed reduced endothelial cell infiltration (**Figure 5D**), consistent with our observation of reduced *in vitro* migration and angiogenesis of HUVECs affected by ZVI-NP (**Figure 2E-I**). Furthermore, mRNA expression levels of NRF2 target genes including *SCL7A11*, *GPX4*, *SLC40A1* and *AKR1* family genes were downregulated in ZVI@Ag NPs-treated xenografts as compared to the control group (**Figure 5E**). Similar results were observed in ZVI@CMC NPs-treated xenografts model (Figure S5K). These findings together indicated that ZVI-NP treatment effectively reduced tumor growth and suppressed cytoprotective NRF2-regulated transcriptional regulatory functions *in vivo*.

To verify whether ZVI-NP treatment-induced anti-tumor effects were attributed to downregulation of the NRF2 pathway and ferroptotic lipid peroxidation *in vivo*, overexpression of NRF2 or Liproxstatin-1 treatment were conducted in H460 subcutaneous xenograft model treated with or without ZVI@CMC NPs. As shown in **Figure 5F-H**, tumor volume, tumor size and tumor weight were significantly reduced after ZVI@CMC NPs treatment as compared to PBS control. Importantly, the addition of Liproxstatin-1 or overexpression of NRF2 significantly attenuated the anti-tumor growth effects of ZVI@CMC NPs, confirming that NRF2 degradation is involved in ZVI@CMC NPs-induced ferroptotic cell death signaling *in vivo*.

To further validate the *in vivo* anti-metastatic effect of ZVI-NP, we first conducted an experimental metastasis model. H460 lung cancer cells were i.v. injected into tail-vein of NOD/SCID mice and then treated with 25 mg/kg of ZVI@CMC or PBS control. As shown by the H&E-stain in **Figure 5I-K**, significantly larger and more lung tumor nodules were observed in control mice as compared to those in the ZVI@CMC-treated mice sacrificed on day 21. We further employed spontaneous lung metastasis model to investigate the anti-metastasis effect of ZVI-NP* in vivo*. We observed large tumor nodules in lung of the control mice (**Figure 5L-N**). In contrast, ZVI@Ag NPs treatment reduced the metastatic tumor nodules, suggesting that ZVI@Ag NPs could suppress metastatic tumors in the lung. These *in vivo* results indicated that ZVI-NP could downregulate the NRF2 pathway and induce ferroptosis in cancer cells, while effectively suppressing both tumor growth and distant metastasis without apparent adverse effects *in vivo*.

### ZVI-NP modulates immune cell profile in mouse model *in vivo*

**Figure 6:**
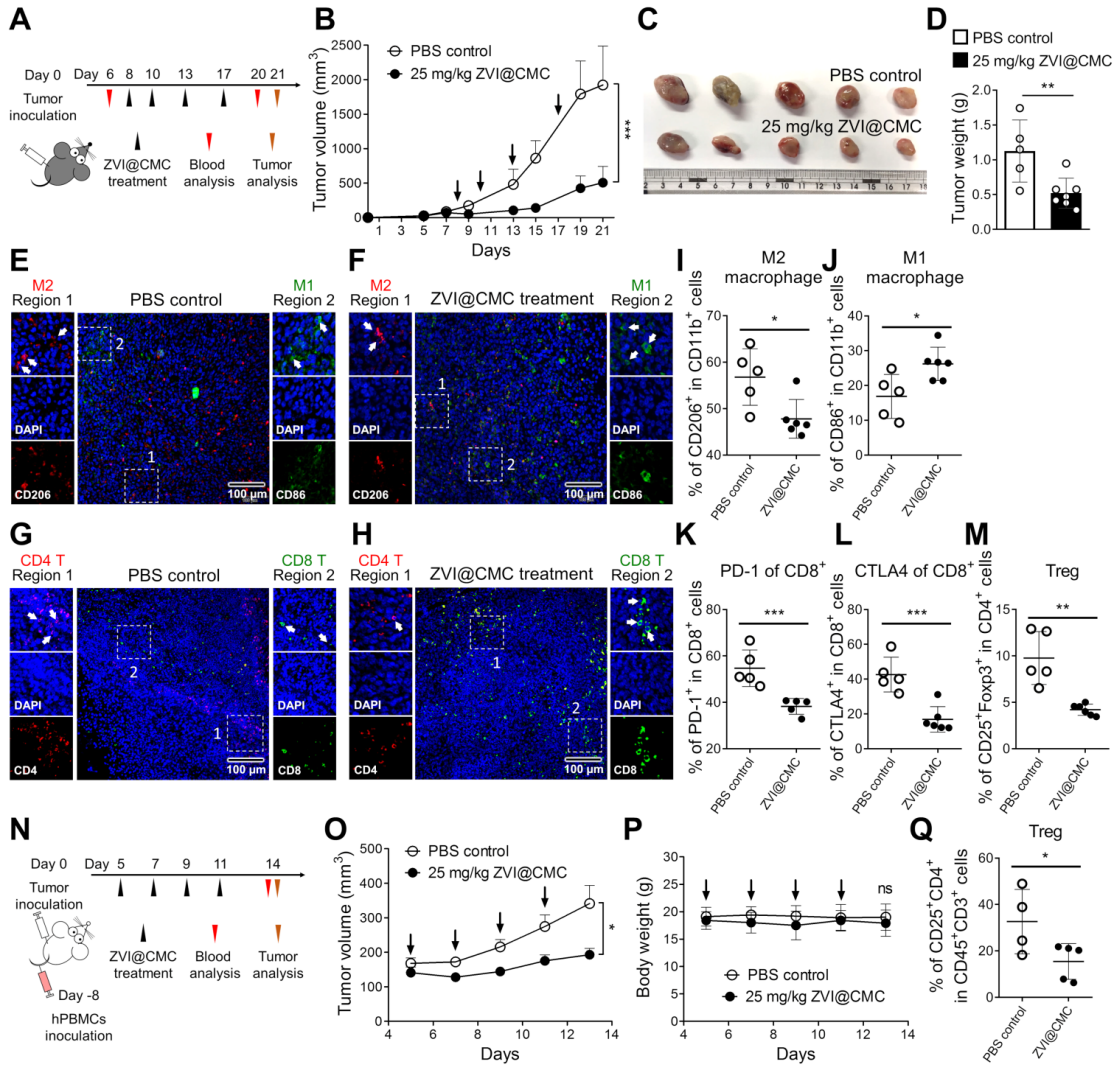
ZVI-NP treatment inhibited tumor growth and modulated immune cell profile *in vivo*. **A,** Schematic illustration of dosing regimens of ZVI@CMC in immunocompetent C57BL/6 mice bearing LLC allografts. C57BL/6 mice were treated with i.v. injection of ZVI@CMC (25 mg/kg) or PBS twice a week as indicated by arrows (*n* = 5 per group). **B,** The changes in tumor volume over experimental period. **C** and **D,** The representative images of the dissected tumor (C) and the quantification of tumor weight (D) were measured at the endpoint of the experiment. **E-H,** Immunofluorescent microscopy images of tissue sections were stained with antibodies against mouse CD86 (*green*) and CD206 (*red*) for observation of tumor-associated macrophages (E and F) and antibodies against mouse CD8 (*green*) and CD4 (*red*) to observe infiltrating T cells (G and H). Scale bar: 100 µm. **I-M,** Flow cytometry analysis of the tumor-associated macrophages (I and J) and infiltrating T cells (K-M) in endpoint tumors. **N,** Schematic illustration of dosing regimens of ZVI@CMC in hPBMC reconstituted ASID mice bearing H460 xenografts. Mice were treated with ZVI@CMC (25 mg/kg) or PBS by i.v. injection on every other day as indicated by arrows (*n* = 5 per group). **O** and **P,** The tumor volume (O) and the body weight (P) were measured during experiment. **Q,** Flow cytometry analysis of Tregs in endpoint tumors. Data were mean ± s.e.m. ns: non-significant; *, *p* < 0.05; **, *p* < 0.01; ***, *p* < 0.001.

The observations of cancer-specific cytotoxicity while sparing the normal lung cells, BMDMs and lymphocytes upon ZVI-NP treatment (**Figure 1D-G**) encouraged the hypothesis that ZVI-NP may modulate immunity *in vivo* in addition to their endogenous anti-tumor efficacy. ZVI@CMC was used to explore the immunomodulation functions because of its biocompatible coating and mass-producibility potential. We established a syngeneic mouse model by subcutaneous injection of LLC cells into immunocompetent C57BL/6 mice and observed the tumor growth and immune cell profile with or without i.v. injection of ZVI@CMC (**Figure 6A**). As shown in **Figure 6B-D**, tumor growth measured by tumor volume, tumor image and tumor weight was significantly reduced after ZVI@CMC NPs treatment as compared to the PBS control. Additionally, body weight, blood biochemistry analysis and H&E-stained tissue sections of major organs showed no obvious difference between the control and ZVI@CMC NPs-treated groups (Figure S6A-C), indicating that ZVI@CMC inhibited tumor growth in immunocompetent mice without apparent adverse effects.

Subsequently, we collected the endpoint LLC allografts from the mice to analyze tumor-infiltrating macrophages and T cells by immunofluorescence microscopy (**Figure 6E-H**) and flow cytometry analysis (**Figure 6I-M**). The immunofluorescence images showed that ZVI@CMC treatment increased the infiltration of anti-tumor M1 macrophages (CD86^+^) and cytotoxic T cells (CD8^+^) in the center of tumor lesions (*region 2,* **Figure 6F, H**) as compared to the control group which showed predominantly peri-tumor localization of M1 macrophages and CD8+ T cells (*region 2,* **Figure 6E, G**). In addition, flow cytometry analysis demonstrated that ZVI@CMC treatment decreased the proportion of M2-like macrophages (**Figure 6I**) but increased that of M1-like macrophages (**Figure 6J**) among tumor associated macrophages. Also, among tumor-infiltrating CD8^+^ T cells, the proportion of PD-1^+^ cells and that of CTLA4^+^ cells were decreased by ZVI@CMC treatment (**Figure 6K-L**). Concomitantly, the proportion of tumor-infiltrating regulatory T cells (Tregs, CD25^+^ FoxP3^+^), a subset of CD4^+^ T cells that have pro-tumor influences, was reduced after ZVI@CMC treatment (**Figure 6M**). Moreover, similar results were observed in the circulating blood of the treated mice (Figure S6D-H), indicating that ZVI@CMC-induced anti-tumor immunity was systemic.

To further investigate how ZVI@CMC modulated the human immune system, we used the advanced severe immunodeficiency (ASID) mice implanted with human peripheral blood mononuclear cells (hPBMCs). After hPBMC engraftment, hPBMC mice bearing subcutaneous H460 tumor xenografts were treated with i.v. injection of ZVI@CMC (25 mg/kg) (**Figure 6N**). As shown in **Figure 6O**, tumor volume was significantly reduced after ZVI@CMC treatment as compared to the PBS control. Body weight, H&E-stained tissue sections of major organs, and blood biochemistry analysis of the mice showed no obvious difference between PBS control and ZVI@CMC NPs treatment groups (**Figure 6P**; Figure S6I-J). Notably, flow cytometry analysis showed that ZVI@CMC treatment decreased the proportion of Tregs among tumor-infiltrating T cells (**Figure 6Q**). Collectively, these results suggested that ZVI-NP treatment could modulate human immunity and provide anti-tumor efficacy *in vivo*.

### ZVI-NP stimulates macrophage and lymphocyte immunity *ex vivo*

**Figure 7:**
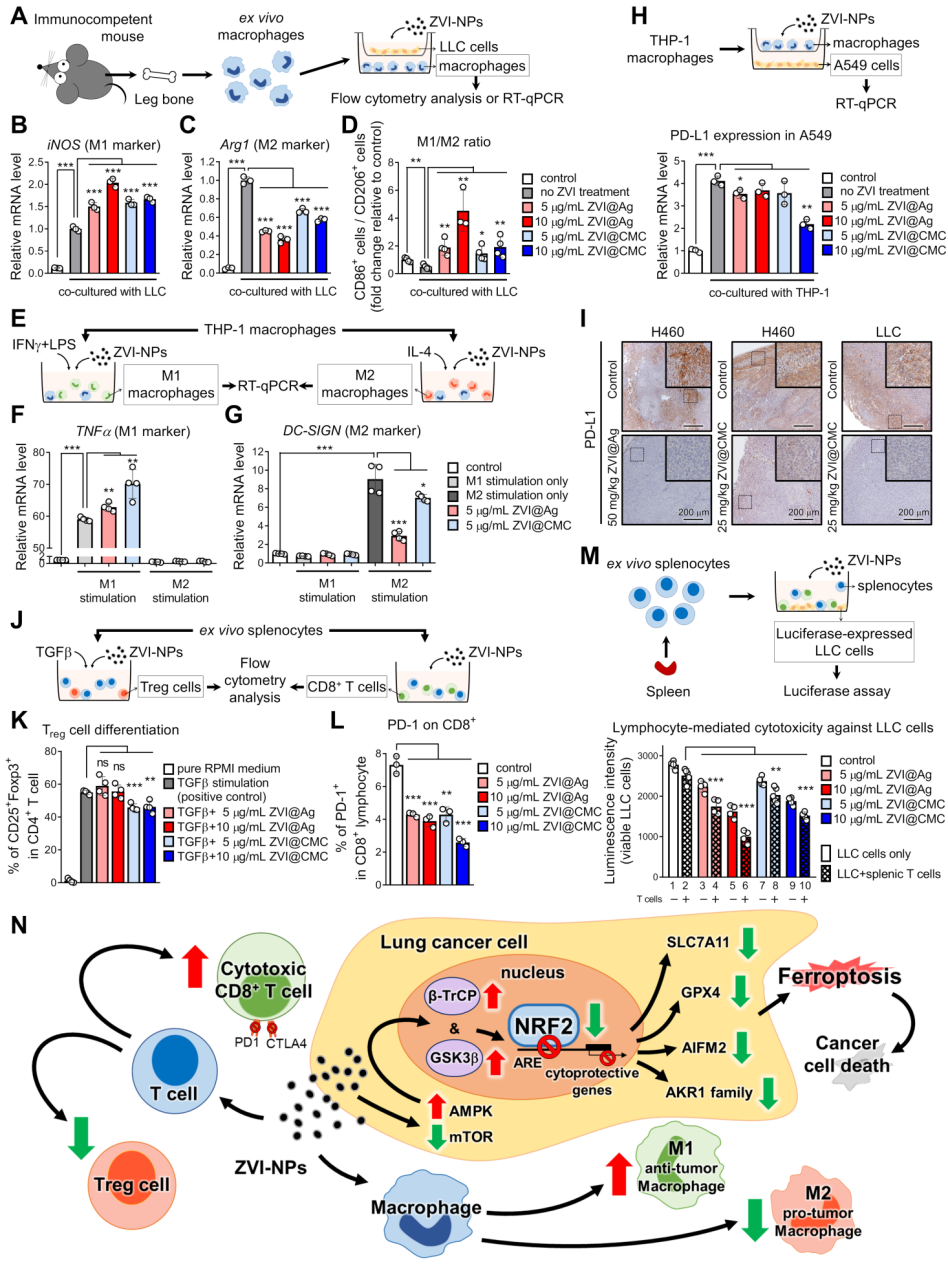
ZVI-NP modulated immune cell profile* in vitro* and *ex vivo*.** A,** Scheme of co-culture system using *ex vivo* macrophages and LLC cells. **B** and **C,** Expressions of M1 associated gene (*iNOS2*) (B) and M2 associated gene (*Arginase-1*) (C) were measured by RT-qPCR in BMDMs treated with ZVI-NPs while co-cultured with LLC cancer cells. The expression levels of mRNA were normalized to *β-actin*. **D,** The percentage of CD86^+^/CD206^+^ (M1/M2) macrophage was determined by flow cytometry. **E,** Scheme of polarization reprogramming of macrophages treated with ZVI-NPs. **F** and **G,** Expressions of M1 associated gene (*TNFα*) (F) and M2 associated gene (*DC-SIGN*) (G) were measured by RT-qPCR in THP-1 macrophages treated with ZVI-NPs while stimulated using IFN-γ plus LPS for M1 polarization and IL-4 for M2 polarization. The expression levels of mRNA were normalized to *β-actin*. **H,** Scheme of co-culture system using THP-1 and A549 cells (*upper*). Gene expression of PD-L1 was measured in A549 cells that were co-cultured with THP-1 cells (*lower*). **I,** The expression of PD-L1 was measured by immunohistochemistry staining in H460 xenografts and LLC allografts. **J,** Scheme of *ex vivo* splenocytes treated with ZVI-NPs. **k,** The percentage of Treg cell differentiation of ZVI-NP-treated splenic T cells with 2 *n*g/mL TGF-β stimulation was analyzed by flow cytometry. **L,** The percentage of PD-1^+^ in CD8^+^ T cells treated with ZVI-NPs was analyzed by flow cytometry. **M,** Luciferase-LLC cells (LLC-luc) were mixed and cultured with splenic lymphocytes at 1:10 ratio, and then treated with ZVI-NPs for 24 h (*upper*). Cancer cell viability was measured by luciferase assay (*lower*). **N,** The model of dual synergistic anti-cancer activities of ZVI-NPs. Data were mean ± s.e.m. (*n* = 3). ns: non-significant; *, *p* < 0.05; **, *p* < 0.01; ***, *p* < 0.001.

To verify whether cancer cell-induced polarization of macrophages can be reprogrammed by ZVI-NP, *ex vivo* isolated BMDMs co-cultured with LLC cells were treated with ZVI-NPs (**Figure 7A-C**). The RT-qPCR results demonstrated that both ZVI@Ag and ZVI@CMC NPs were able to enhance the expression of M1 marker *iNOS* but reduced the level of M2 marker *Arginase-1* (*Arg1*) in BMDMs under the cancer cell co-culture condition. Consistently, flow cytometry analysis indicated that the ratio of M1-like/M2-like macrophages among the co-cultured BMDMs was increased after ZVI-NP treatment (**Figure 7D**). To further determine the effects of ZVI-NP treatment on macrophage polarization, THP-1 macrophages were treated with ZVI-NPs while stimulated with IFN-γ plus LPS for M1 polarization and IL-4 for M2 polarization (**Figure 7E-G**). RT-qPCR results revealed that ZVI-NP treatment promoted the M1 polarization induction-derived overexpression of *TNF-α*, while attenuated the expression of the M2 polarization gene *DC-SIGN*. These data suggested that ZVI-NP treatment modulated macrophage polarization toward M1 phenotype under the cancer co-culture system or macrophage polarization stimulation.

Further, we examined whether the PD-L1 expression in A549 cancer cells co-cultured with THP-1 macrophages was affected by ZVI-NP treatment (**Figure 7H**). RT-qPCR results showed that the level of PD-L1 in A549 cells was significantly increased after co-cultured with THP-1 macrophages, and the PD-L1 overexpression could be attenuated by ZVI-NP treatment. Importantly, the IHC staining of allograft and xenograft tumor tissues showed that PD-L1 expression was dramatically downregulated in ZVI-NP-treated groups (**Figure 7I**). Together, these findings suggested that ZVI-NP treatment improved anti-cancer immunoresponses by modulating macrophage polarization toward M1 phenotype and inhibiting the expression of PD-L1 on cancer cells.

Next, we investigated the T cell signaling and expression of inhibitory immune checkpoint protein PD-1 in splenic lymphocytes after ZVI-NP treatment (**Figure 7J**). We first used TGF-β stimulation to induce Treg differentiation in *ex vivo* isolated splenic lymphocytes. Flow cytometry analysis showed that ZVI@CMC NPs treatment decreased the proportion of Tregs (**Figure 7K**). Notably, the results of flow cytometry analysis demonstrated that both ZVI@Ag and ZVI@CMC NPs reduced the proportion of PD-1^+^ cells among CD8^+^ lymphocytes (**Figure 7L**). Furthermore, luciferase-expressing LLC cell line was used for measuring lymphocyte-mediated cytotoxicity against cancer cells (**Figure 7M**). We observed that the luminescence intensity was lower in lane 2 as compared to lane 1, indicating that the population of viable LLC cells was reduced after co-culture of LLC cells with splenic lymphocytes. In particular, the lymphocyte-mediated cytotoxicity was further promoted by both ZVI@Ag and ZVI@CMC NPs (*lanes* 4, 6, 8, 10, **Figure 7M**). Collectively, these results suggested that ZVI-NP treatment could enhance lymphocytic immunity and promote M1-type macrophages to elicit anti-tumor properties *in vitro*.

## Discussion

In this study, we discovered the dual anti-cancer activities of ZVI-NP through inducing cancer cell ferroptotic death and modulating cancer microenvironment favorable to anti-tumor immune responses. ZVI-NP triggered ferroptosis selectively in cancer cells by suppressing NRF2-mediated cytoprotection program, which was attributed to the ZVI-NP-induced disruption of AMPK/mTOR signaling and activation of GSK3β/β-TrCP-dependent degradation system. Furthermore, ZVI-NP prominently modulated macrophages' polarization from immunosuppressive M2 phenotype to anti-tumor M1 phenotype and increased cytotoxic function of CD8^+^ T cells as well as reduced Treg proportion to augment anti-tumor immunity in our *ex vivo* and *in vivo* models (**Figure 7N**).

KEAP1 is a well-known E3-ligase of NRF2 31 and is deficient in A549 and H460 cells. We found that aberrant NRF2 expression was suppressed upon ZVI-NP treatment *via* activation of the alternative E3-ligase β-TrCP in conjunction with the phosphorylation of GSK3β. In addition, ZVI-NP treatment decreased mTOR phosphorylation at the active site and manifested an inverse correlation between mTOR and GSK3β/β-TrCP degradation signaling in ZVI-NP-treated cells. This finding is quite important, as several studies have reported that mTOR negatively regulates the GSK3β-dependent pathways 35-37. Interestingly, it has been demonstrated that iron overload promotes AMPK phosphorylation 38. Here, we provide the first evidence that AMPK presents a mechanistic link between ZVI-NP-induced iron overload and NRF2 degradation. Our results elucidated a novel mechanism whereby ZVI-NP disrupts redox balance and induces ferroptosis of lung cancer cells by triggering AMPK/mTOR signaling to promote GSK3β/β-TrCP degradation of NRF2.

It is worth to mention that *AIFM2* has recently been identified as a potent ferroptosis-resistance factor 29, 30. Our results provide first ChIP binding evidence that NRF2 bound at transcriptional target gene *AIFM2*, and ZVI-NP treatment reduced the NRF2-mediated expression of *AIFM2*. In fact, transient NRF2 activation can protect cell from external stress; however, persistent NRF2 activation in cancer cells (known as NRF2 addiction) confers therapeutic resistance and aggressive tumorigenicity 39, 40. Hence, ZVI-NP could be a promising anti-cancer strategy for NRF2-addicted cancers.

Recently, targeting iron homeostasis in immune cells has received substantial interest as it involves anti-cancer immunity. M2 macrophages contain lower intracellular iron and promote tumor growth, while M1 macrophages are “iron-retaining” with proinflammatory activity to limit tumor progression and even to kill tumor cells 41, 42. Iron metabolism also plays an important role in T cell activation and proliferation. T cell activation can be boosted by iron-dextran NPs 43. A recent study demonstrated that iron promotes the production of pro-inflammatory cytokines not only in anti-tumor T helper 1 cells but also in T helper 17 cells, a subtype of T cells performing as the antagonist of Treg cells 44. Our results showed that ZVI-NP treatment decreased the proportion of Treg cells and enhanced the cytolytic activity of CD8^+^ lymphocytes. Taken together, these studies and our results reveal that iron regulation plays a significant role in anti-cancer responses of both macrophages and T cells.

Notably, cystine/glutamate antiporter xCT (encoded by *SLC7A11*), mediating intracellular redox balance and preventing ferroptosis, is dispensable for T cell proliferation and anti-tumor immunity* in vivo* 45. This suggests that ZVI-NP-induced downregulation of *SLC7A11* may have an impact on tumor growth but not on T cells. Indeed, ZVI-NP did not adversely affect the proliferation of lymphocytes and BMDMs in our *ex vivo* culture system. Previously, iron nanoparticle was reported to overcome tumor hypoxia through Fenton reaction and induce immunostimulatory tumor microenvironment by increasing the infiltration of effector T cells 46. Of note, nanoparticle-mediated ROS generation in macrophages can reprogram their polarization toward M1 type and enhance their antigen presentation and T cell priming 47. Besides, ROS-induced endosomal lipid peroxidation promotes antigen presentation in dendritic cells 48. Taken together, ZVI-NP-induced ROS production in these antigen presenting cells may boost their presentation of antigen to T cells, hence augmenting the activation of cytotoxic T cells and the anti-cancer responses.

In addition, ferroptotic cells can release damage-associated molecular patterns (DAMPs) into the surrounding microenvironment and thus initiate non-infectious pro-inflammatory response of macrophages 49-51. A recent study reported that DAMPs released from ferroptotic cancer cells can promote the maturation of dendritic cells and result in an adaptive immune response 52. Accordingly, ferroptotic DAMPs could partly account for how ZVI-NP shapes an anti-tumor microenvironment. More functional analyses are needed to clarify the role of ferroptosis-related DAMPs in ZVI-NP-induced anti-tumor immunity. Interestingly, immunotherapy and radiotherapy have recently been demonstrated to activate CD8^+^ T cells and to modulate tumor ferroptosis through IFNγ pathway 53, 54. The synergistic effect of ZVI-NP and other cancer therapeutics such as radiotherapy or immunotherapies may worth further investigation.

Our biodistribution analysis of ZVI-NPs showed the iron levels in liver and heart are limited while they were mainly retained in the tumor and lung tissues for more than 120 h. By contrast, previous studies using other iron-based NPs showed much higher concentration of iron species accumulated in the liver, spleen, kidneys, and other tissues 55-57. Unlike normal blood vessels, the higher permeability and irregular shape of tumor vasculature allow enhanced permeation and retention effect (EPR) of certain size range of NPs to be preferentially trapped within tumor microenvironment for effective nanomedicine delivery 58. Our observations of preferred lung tissue localization in synergy with EPR suggested that ZVI-NP treatment could be an effective strategy against lung cancer.

Our previous works demonstrated that bared-ZVI NPs can induce cancer-selective cytotoxicity 3, 5, and it was suggested that ZVI instead of CMC or Ag plays major roles in ferroptosis while the surface coating modulates the speed of intracellular ZVI conversion 59. In order to enhance cellular uptake, either active targeting by surface ligands conjugation 22 or passive targeting *via* surface charge modifications 60, 61 have been explored. It was reported that positively charged NPs are rapidly uptaken by smaller-sized tumors, but negatively charged NPs penetrated deeper into larger-sized tumors and appreciably escalate its clinical utility in cancer management 60. This may explain the ability of our negatively charged ZVI-NPs to penetrate deep into tumor tissues and generate burst release of iron ions to induce ROS surge followed by subcellular organelles damage and ferroptosis 10.

Since cancer stem cells are associated with chemoresistance and relapse of the disease 62, and angiogenesis is required for tumor growth and metastasis 63, concentrated accumulation of NPs focused on targeting cancer stem cells and angiogenic vessels is of great importance. Interestingly, we discovered that ZVI-NPs also showed effectiveness in anti-cancer stemness and anti-angiogenesis. ZVI-NPs presented potent efficacy in suppressing tumor sphere formation and expressions of cancer stemness-associated genes. ZVI-NPs also actively attenuated cancer cell migration and invasion. We also showed dose-dependent inhibition of endothelial cells tubule formation and expressions of pro-angiogenic genes. Further augmentation of ZVI-NPs to accumulate inside tumor tissues could be engineered by ameliorating the physical, chemical properties and assembly of active targeting moieties on the particles.

In conclusion, this study identifies a dual mechanism of anti-cancer activities of ZVI-NP that spares non-malignant cells. The first mechanism involves enhanced GSK3β/β-TrCP-dependent degradation of NRF2 through activation of the AMPK/mTOR signaling pathway, and thereby triggering ferroptosis selectively in lung cancer cells. The second mechanism is through activating anti-tumor immune responses. It involves both modulation of macrophage polarization toward anti-tumor M1 phenotype and boosting the cytolytic activity of CD8^+^ lymphocytes as well as decreasing the proportion of Treg cells. Through understanding of the molecular mechanism, we proposed that NRF2 or associated proteins may serve as biomarkers for lung cancer or NRF2-addicted cancer patients that may benefit from ZVI-NP treatment. In addition, ZVI@CMC having biocompatible coating and mass producibility may exert the potential for new advanced cancer therapy. These results provide an insight into development of novel anti-cancer precision nanomedicine that synergistically targets both cancer cells and tumor microenvironment.

## Supplementary Material

Supplementary figures and tables.

## Abbreviations

ASID: advanced severe immunodeficiency
BMDM: bone-marrow-derived macrophage
ChIP: chromatin immunoprecipitation
CM: condition medium
DAMP: damage-associated molecular pattern
HUVEC: human umbilical vein endothelial cell
hPBMC: human peripheral blood mononuclear cell
IF: immunofluorescence
IHC: immunochemistry
i.v.: intravenous
i.p.: intraperitoneal
mtROS: mitochondrial reactive oxygen species
NP: nanoparticle
OCR: oxygen consumption rate
ROS: reactive oxygen species
Treg: regulatory T cell
ZVI: zero-valent-iron
ZVI@Ag: silver coated ZVI-NP
ZVI-CMC: carboxymethylcellulose coated ZVI-NP
