# Supplementary material for: An innovative NRF2 nano-modulator induces lung cancer ferroptosis and elicits an immunostimulatory tumor microenvironment
Source: Theranostics. 2021 May 13;11(14):7072–91. doi: 10.7150/thno.57803 (PMC8171079; doi:10.7150/thno.57803)
Supplement: Supplementary file 1 — Supplementary figures and tables. [file thnov11p7072s1.pdf]

Supplementary Information

An innovative NRF2 nano-modulator induces lung cancer ferroptosis and elicits an immunostimulatory tumor microenvironment

Chih-Hsiung Hsieh, Hung-Chia Hsieh, Fu-Hsuan Shih, Pei-Wen Wang, Li-Xing Yang, Dar-Bin Shieh, and Yi-Ching Wang

<u>Inventory of supplementary information</u>

Supplementary Figures and Tables

Figure S1 is related to Figure 1.

Figure S2 is related to Figure 2.

Figure S3 is related to Figure 3.

Figure S4 is related to Figure 4.

Figure S5 is related to Figure 5.

Figure S6 is related to Figure 6.

Table S1 is related to Materials and Methods.

Table S2 is related to Materials and Methods.

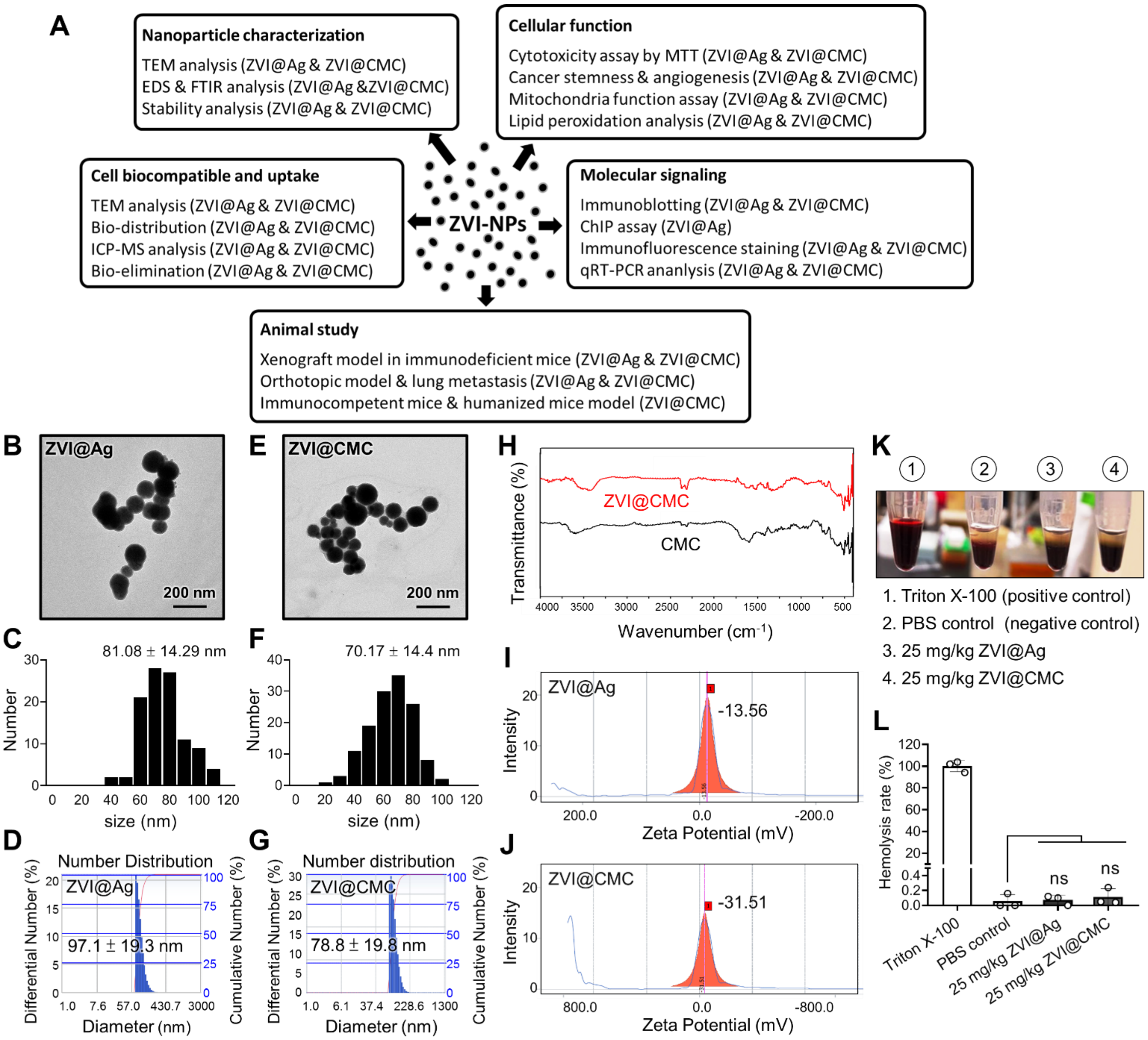

Figure S1. The properties of ZVI-NPs. **A,** Schematic of ZVI-NP application in each experiment. **B-D,** The characterization analysis of ZVI@Ag. (B) Ultrastructure of ZVI@Ag observed under TEM at 40000X magnification. (C) The histogram of particle size quantized from TEM observation. (D) The number distribution was determined by dynamic light scattering analysis. **E-G,** The characterization analysis of ZVI@CMC. (E) Ultrastructure of ZVI@CMC observed under TEM at 40000X magnification. (F) The histogram of particle size quantized from TEM observation. (G) The number distribution was determined by dynamic light scattering analysis. **H,** FTIR spectra of the

carboxymethyl cellulose (CMC) and ZVI@CMC showed successful coating of the polymer to the nanoparticles. **I** and **J,** The zeta potential of ZVI@Ag (I) and ZVI@CMC (J). **K** and **L,** Haemolysis effects determined according to ISO10993-4. (K) Image of samples after centrifugation at 3000 rpm for 5 min. (L) Haemolytic activities of ZVI@Ag and ZVI@CMC incubated with human RBCs for 1 h. Data were mean ± s.e.m. ns: non-significant; *, $p < 0.05$; **, $p < 0.01$; ***, $p < 0.001$.

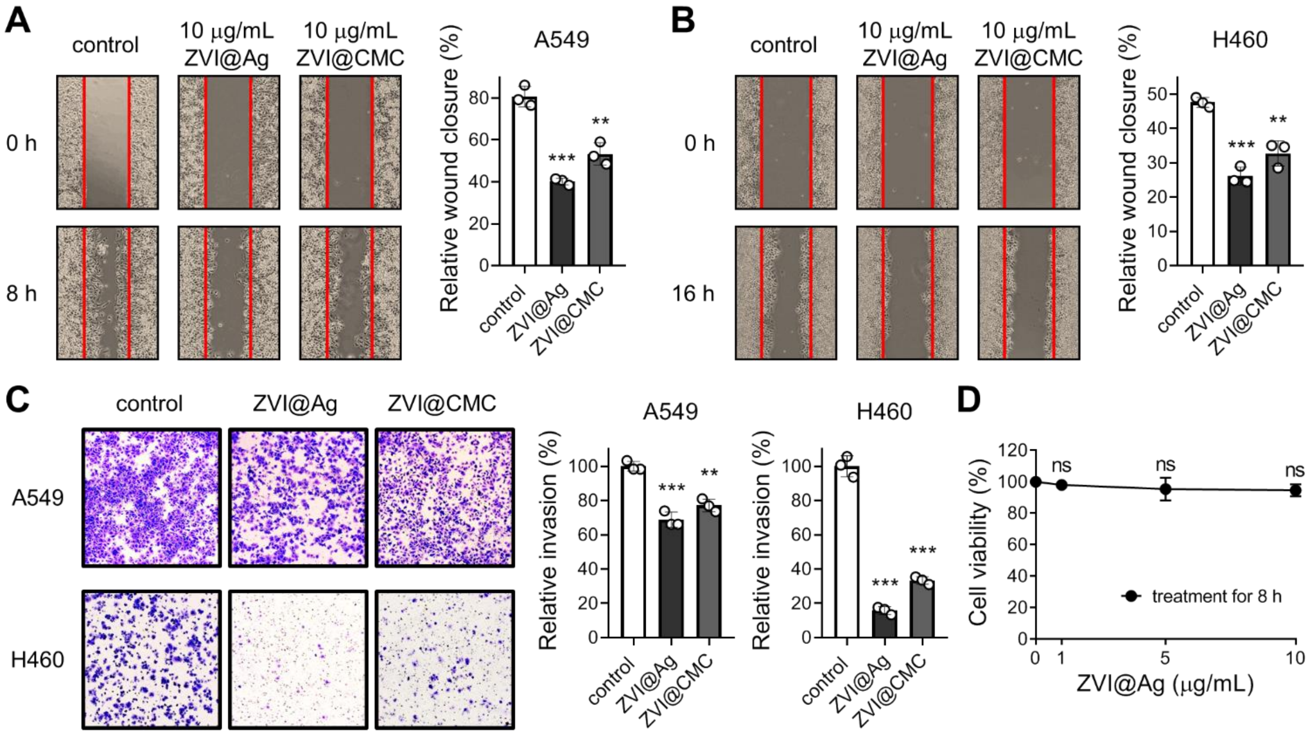

Figure S2. The effects of ZVI-NPs on migration and invasion abilities of cancer cells and viability of HUVECs. **A** and **B,** The wound healing migration ability of A549 (A) and H460 (B) cells treated with or without ZVI-NPs. **C,** The transwell migration ability of A549 and H460 cells treated with or without ZVI-NPs for 16 h. **D,** The effect of ZVI@Ag on HUVECs viability determined by MTT assay after treatment for 8 h. Data were mean ± s.e.m. ns: non-significant; *, $p < 0.05$; **, $p < 0.01$; ***, $p < 0.001$.

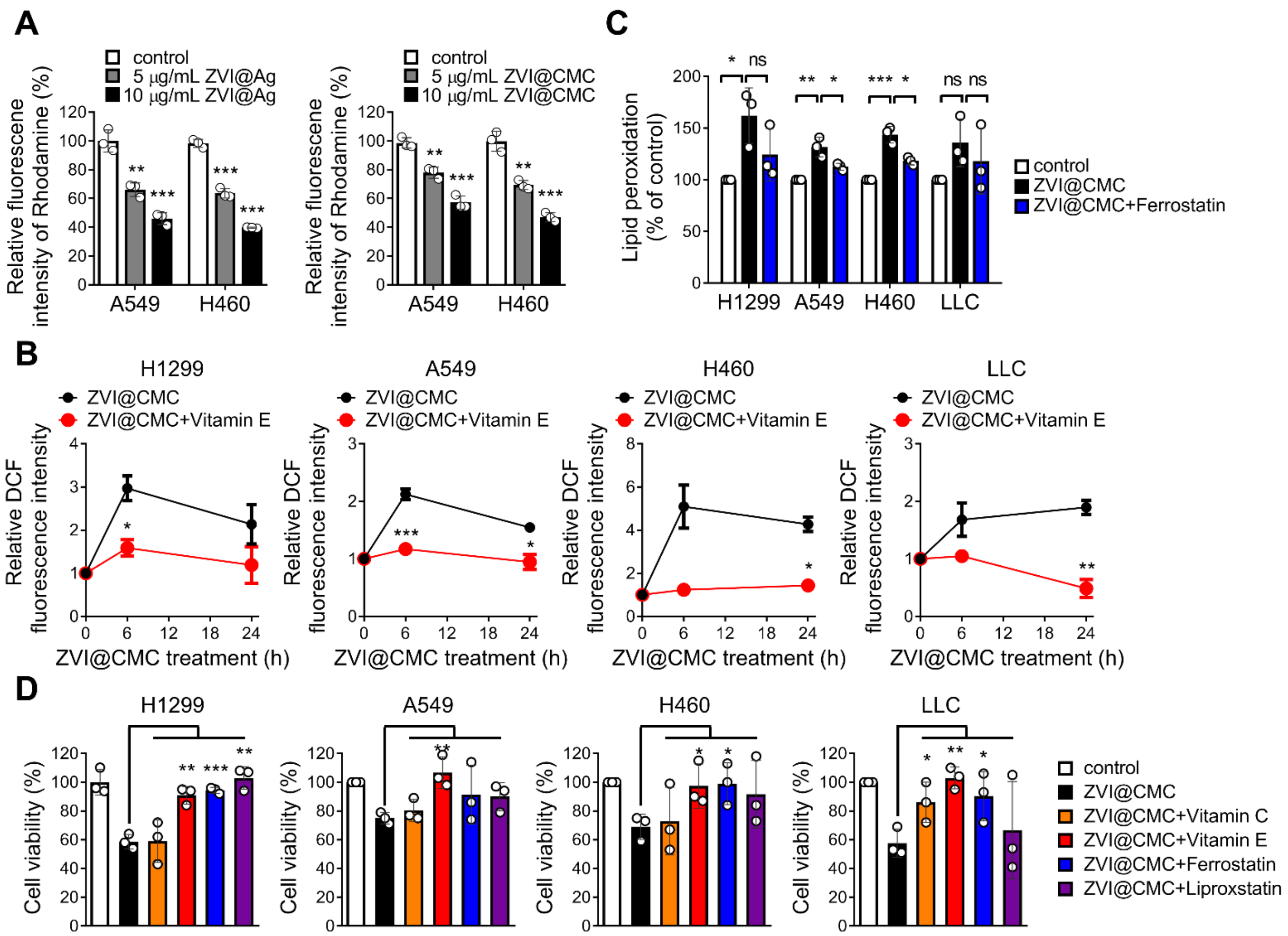

Figure S3. ZVI-NPs caused oxidative stress and lipid peroxidation *in vitro*. **A,** Mitochondrial membrane potential was analyzed by flow cytometry analysis of Rhodamine 123 after ZVI-NPs treatment. **B,** Intracellular ROS level was measured by flow cytometry after ZVI@CMC NPs (5 μg/mL) treatment with or without Vitamin E (100 μM). **C,** Analysis of lipid peroxidation was measured by flow cytometry. Cells were treated with ZVI@CMC NPs (5 μg/mL) with or without Ferrostatin (10 μM) pre-treatment. **D,** Cell viability was determined after co-treatment with ZVI@CMC NPs (10 μg/mL) and Vitamin C (100 μM) for 24 h, Vitamin E (100 μM) for 24 h, Ferrostatin (10 μM) for 48 h, or Liproxstatin (10 μM) for 48 h. Data were mean ± s.e.m. ns: non-significant; *, $p < 0.05$; **, $p < 0.01$; ***, $p < 0.001$.

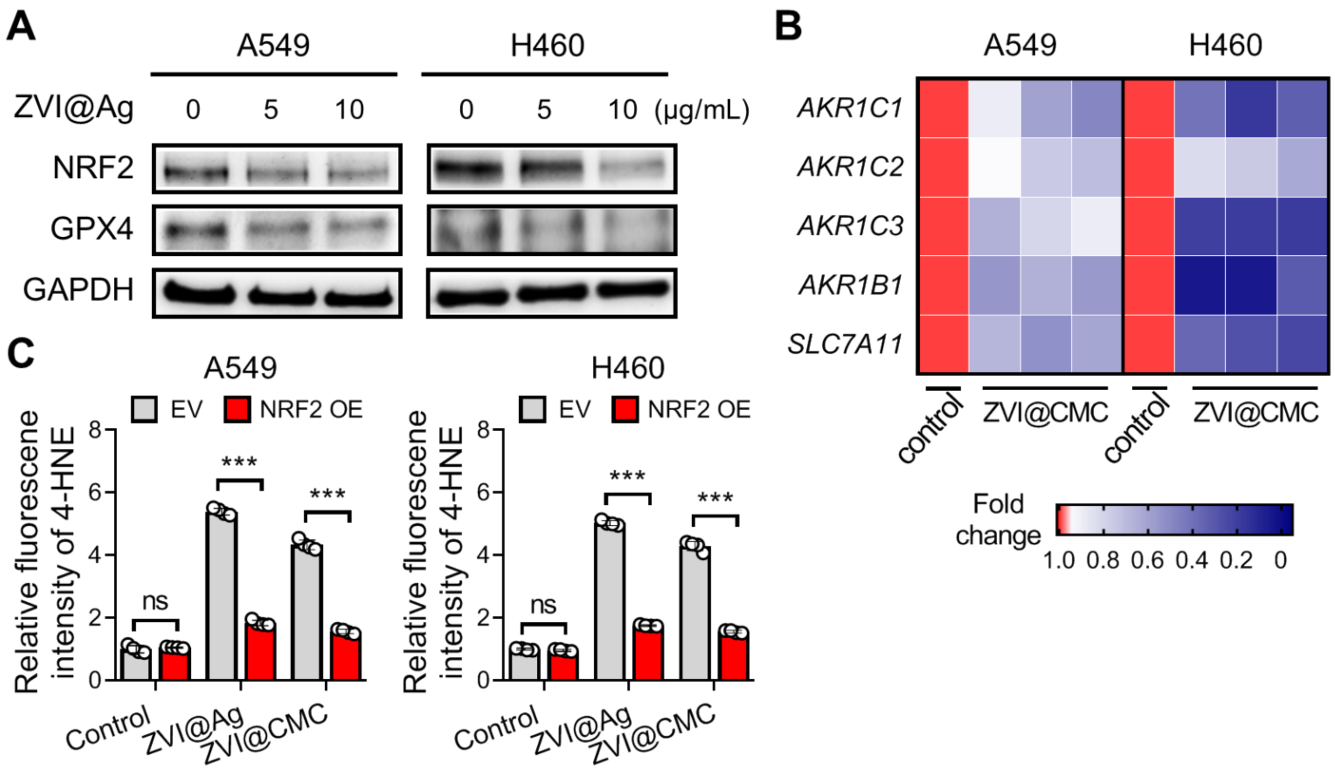

Figure S4. ZVI-NPs inhibited NRF2-regulated antioxidant activity *via* enhancement of GSK3β/β-TrCP degradation pathway. **A,** Immunoblotting for NRF2 and GPX4 in cells treated with ZVI@Ag NPs at the indicated doses for 24 h. GAPDH was used as internal control. **B,** mRNA expression of NRF2 downstream genes was measured by RT-qPCR after ZVI@CMC NPs treatment in A549 and H460 cells. Heat map colors reflect the downregulation of the mRNA levels of these genes compared to untreated control. **C,** The intracellular level of lipid peroxidation by analysis of 4-HNE in ZVI-NP-treated cells with or without NRF2 overexpression. Data were mean ± s.e.m. ns: non-significant; *, $p < 0.05$; **, $p < 0.01$; ***, $p < 0.001$.

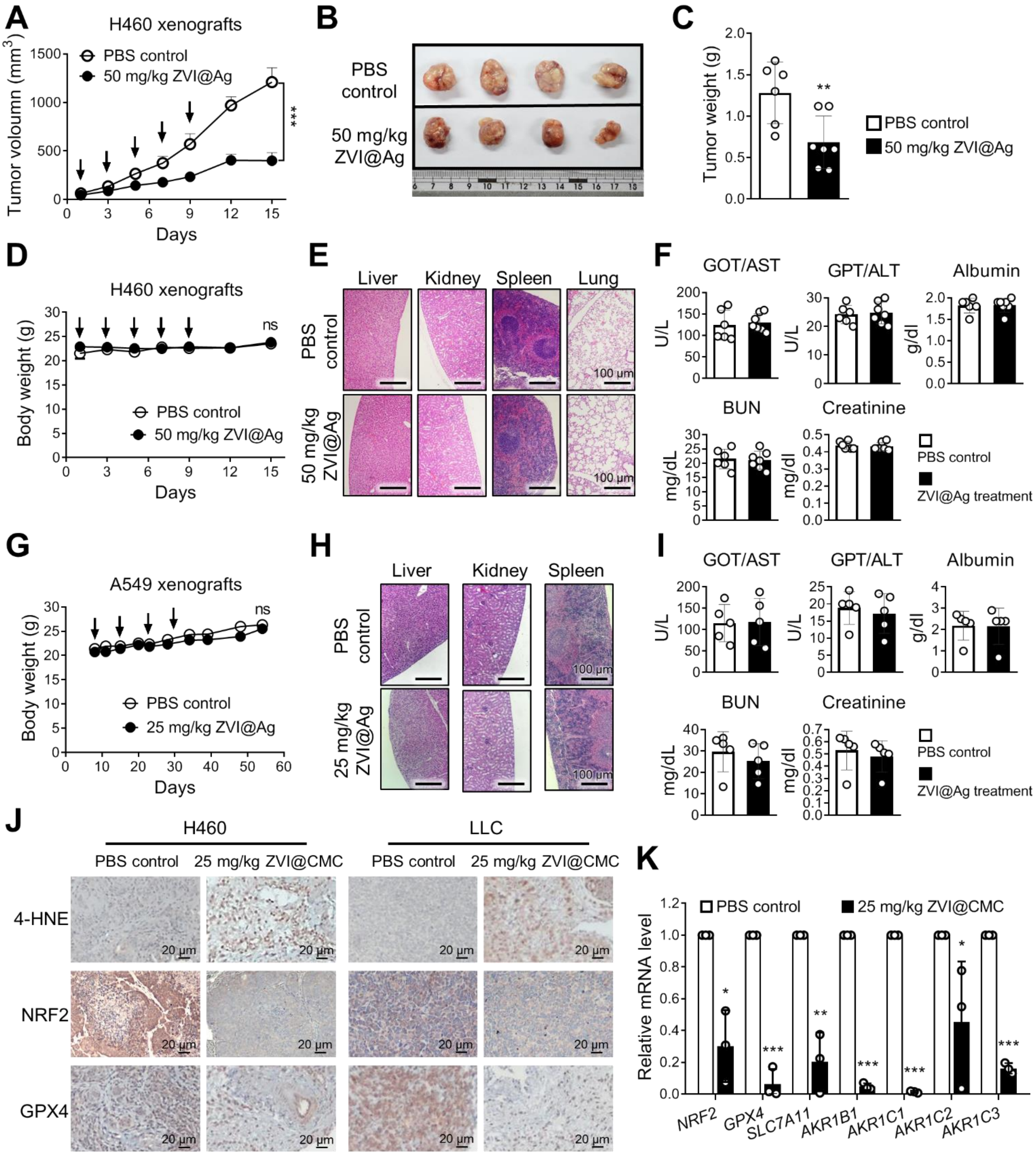

Figure S5. ZVI-NPs inhibited NRF2-regulated antioxidant transcription program *in vivo*. **A-C,** The changes in tumor volume over 15 days of observation period (A), the representative tumor images in the endpoint (B), and the endpoint tumor weight (C) of BALB/c nude mice bearing H460 xenografts treated with 50 mg/kg ZVI@Ag NPs or

PBS by i.p. injection on every other day as indicated by arrows (*n* = 6 for control group, *n* = 7 for ZVI@Ag NPs treated group). **D-F,** The body weight (D), the H&E staining of major organs (E), and the blood biochemistry analysis (F) of BALB/c nude mice bearing H460 xenografts treated with 50 mg/kg ZVI@Ag NPs or PBS by i.p. injection on every other day as indicated by arrows. **G-I,** The body weight (G), the H&E staining of major organs (H), and the blood biochemistry analysis (I) of NOD/SCID mice bearing A549 xenografts treated with 25 mg/kg ZVI@Ag NPs or PBS by i.v. injection once a week as indicated by arrows. **J,** The expression of 4-HNE, NRF2, and GPX4 was measured by immunohistochemistry staining in ZVI@CMC NPs treated H460 xenografts (*left*) and LLC allografts (*right*). **K,** Expression of NRF2 targeting genes was measured by RT-qPCR in H460 xenografts treated with 25 mg/kg ZVI@CMC. Data were mean ± s.e.m. ns: non-significant; *, $p < 0.05$; **, $p < 0.01$; ***, $p < 0.001$.

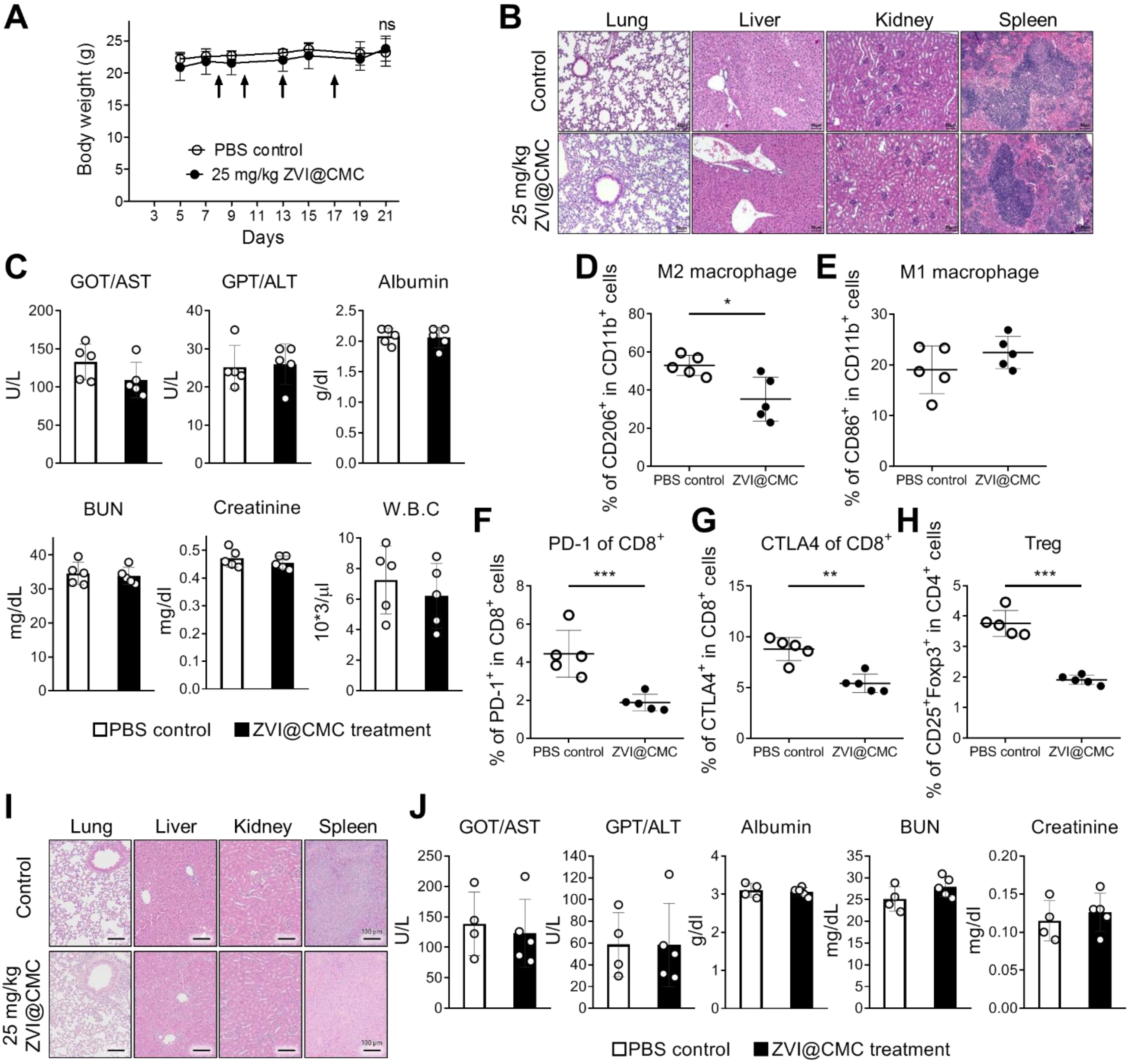

Figure S6. Analysis of body weight, H&E staining of major organs, and circulation blood of mice treated with ZVI@CMC NPs or PBS. **A-C,** The body weight (A), the H&E staining of major organs (B), and the blood biochemistry analysis (C) of C57BL/6 mice bearing LLC allografts treated with i.v. injection of ZVI@CMC NPs (25 mg/kg) or PBS. **D-H,** Flow cytometry analysis of the macrophages (D and E) and T cells (F-H) in circulating blood on day 20. **I** and **J,** The H&E staining of major organs (I) and the blood biochemistry analysis (J) of hPBMC mice bearing subcutaneous H460 tumor xenografts treated with i.v. injection of ZVI@CMC NPs (25 mg/kg) or PBS. Data were mean ± s.e.m. ns: non-significant; *, $p < 0.05$; **, $p < 0.01$; ***, $p < 0.001$.

Supplementary Table S1. The primer sets used in this study.

Gene	species	Primer	Sequences (5'→ 3')	Application[a]	PCR size (bp)	Tm (°C)
β-actin mRNA	human	Forward	GGC GGC ACC ACC ATG TAC CCT	RT-qPCR	202	60
		Reverse	AGG GGC CGG ACT CGT CAT ACT			
SLC7A11 mRNA	human	Forward	ATG CAG TGG CAG TGA CCT TT	RT-qPCR	71	60
		Reverse	GGC AAC AAA GAT CGG AAC TG			
GPX4 mRNA	human	Forward	CAG TGA GGC AAG ACC GAA GTA AA	RT-qPCR	110	60
		Reverse	TGC TTC CCG AAC TGG TTA CAC			
NRF2 mRNA	human	Forward	ACA CGG TCC ACA GCT CAT C	RT-qPCR	83	60
		Reverse	TGT CAA TCA AAT CCA TGT CCT G			
AKR1B1 mRNA	human	Forward	TAC CAT GAG AAG GGC CTG GTG AAA	RT-qPCR	173	60
		Reverse	TCC AGA ATG TTG GTG TCA CTG GGA			
AKR1C1 mRNA	human	Forward	ATT TGC CAG CCA GGC TAG TG	RT-qPCR	179	60
		Reverse	AGA ATC AAT ATG GCG GAA GCC			
AKR1C2 mRNA	human	Forward	AAG TAA AGC TCT AGA GGC CGT	RT-qPCR	86	60
		Reverse	GCT CCT CAT TAT TGT AAA CAT GT			
AKR1C3 mRNA	human	Forward	GGG ATC AAC GAG AGA CAA ACG	RT-qPCR	68	60
		Reverse	AAA GGA CTG GGT CCT CCA AGA			
SLC40A1 mRNA	human	Forward	GCA TGG GTC TTG CTT TCC TTT	RT-qPCR	103	60
		Reverse	AAA ATA CTG AGG ATG GAA CCA CTC A			
Oct4 mRNA	human	Forward	CGA AAG AGA AAG CGA ACC AG	RT-qPCR	157	60
		Reverse	GCC GGT TAC AGA ACC ACA CT			
Sox2 mRNA	human	Forward	ACA ACT CGG AGA TCA GCA	RT-qPCR	183	60
		Reverse	GCA GCG TGT ACT TAT CCT TC			

Nanog mRNA	human	Forward	CTG TGA TTT GTG GGC CTG AA	RT-qPCR	190	60
		Reverse	TCT TCC TTT TTT GCG ACA CTC TT			
Sonic hedgehog mRNA	human	Forward	CCC AAT TAC AAC CCC GAC ATC	RT-qPCR	142	60
		Reverse	TCA CCC GCA GTT TCA CTC CT			
TGFβ mRNA	human	Forward	AAA GCC AGA GTG CCT GAA CAA	RT-qPCR	150	60
		Reverse	AAC AGC ATC AGT TAC ATC GAA GGA			
VEGFA mRNA	human	Forward	TAC CTC CAC CAT GCC AAG TG	RT-qPCR	100	60
		Reverse	TGC GCT GAT AGA CAT CCA TGA			
AIFM2 mRNA	human	Forward	AGG GTT CGC CAA AAA GAC ATT	RT-qPCR	100	60
		Reverse	CAC CAT CTG GTT CTT CAG GTC TAT C			
NDUFF4 mRNA	human	Forward	TAA GAG CAT TCC CAA AGG CAA A	RT-qPCR	100	60
		Reverse	CAT TAT TTT CTC AGC AGT CCA GGT T			
IDH1 mRNA	human	Forward	GTC GTC ATG CTT ATG GGG AT	RT-qPCR	101	60
		Reverse	CTT TTG GGT TCC GTC ACT TG			
ME1 mRNA	human	Forward	TCT TCA TGT TCA TGG GCA AA	RT-qPCR	157	60
		Reverse	GGA TTG CAC ACC TGA TTG TG			
6PGD mRNA	human	Forward	GTC AGT GGT GGA GAG GAA GG	RT-qPCR	96	60
		Reverse	GCC TTG GAA GAT GGT CTT GA			
TNFα mRNA	human	Forward	CCC AGG GAC CTC TCT CTA ATC A	RT-qPCR	116	60
		Reverse	AGC TGC CCC TCA GCT TGA G			
DC-SIGN mRNA	human	Forward	GCA GTC TTC CAG AAG TAA CCGC	RT-qPCR	128	60
		Reverse	GCT CTC CTC TGT TCC AAT ACT GC			
PD-L1 mRNA	human	Forward	GCC AGA AAA GCC TCA TTC GT	RT-qPCR	100	60
		Reverse	TGA ATC TCG AAA CCT CCA GGA A			
β-actin mRNA	mouse	Forward	GGC TCT TTT CCA GCC TTC CT	RT-qPCR	100	60
		Reverse	GTC TTT ACG GAT GTC AAC GTC ACA			

iNOS mRNA	mouse	Forward	TGA CGC TCG GAA CTG TAG CAC	RT-qPCR	98	60
		Reverse	TGA TGG CCG ACC TGA TGT T			
Arg1 mRNA	mouse	Forward	CAT GGG CAA CCT GTG TCC TT	RT-qPCR	103	60
		Reverse	CGA TGT CTT TGG CAG ATA TGC A			
SLC7A11 promoter-ChIP	human	Forward	TTA CTA CTT CTG GAT TGG CTA	ChIP-qPCR	221	60
		Reverse	CTT GTA TTT AAG CGC CTG CC			
AKR1C1 promoter-ChIP	human	Forward	GAA TCC ACC ATC TTG TTG AAA	ChIP-qPCR	150	60
		Reverse	ACA ACT TGC AGT GCC CTG AT			
AIFM2 promoter-ChIP	human	Forward	AGA TGG CTT ATC TTT CGC TGA	ChIP-qPCR	151	60
		Reverse	TCT CCA AGG ATG AGA AAG AGG			

[a] RT-qPCR: Quantitative reverse-transcriptase polymerase chain reaction; ChIP-qPCR: Chromatin-immunoprecipitation qPCR

Supplementary Table S2. The antibodies and their reaction conditions used in this study.

Target	KD	Raised in	Application[a]	Dilution	Source	Catalog no.
NRF2	110	Rabbit	Western blot	1:500	Genetex	GTX103322
			Immunofluorescence	1:1000		
			Immunohistochemistry	1:250		
			ChIP	2 μg		
GPX4	22	Rabbit	Western blot	1:500	Abcam	ab41787
			Immunohistochemistry	1:500		
CD31	-[b]	Rabbit	Immunohistochemistry	1:250	Abcam	ab28364
4-HNE	-[b]	Rabbit	Immunohistochemistry	1:200	Abcam	ab46545
AMPKα	62	Rabbit	Western blot	1:1000	Cell Signaling	5832S
p-AMPKα1/2(T183/T172)	62	Rabbit	Western blot	1:1000	Genetex	GTX63165
mTOR	289	Rabbit	Western blot	1:1000	Cell Signaling	2972S
p-mTOR (S2448)	289	Rabbit	Western blot	1:1000	Genetex	GTX79009
GSK3β	46	Rabbit	Western blot	1:1000	Cell Signaling	9315S
p-GSK3β (Y216)	47	Rabbit	Western blot	1:1000	Abcam	ab75745
β-TrCP	-[b]	Rabbit	Immunofluorescence	1:3000	Cell Signaling	4394s

AKT	60	Rabbit	Western blot	1:1000	Cell Signaling	9272s
p-AKT	60	Rabbit	Western blot	1:500	Cell Signaling	4060s
GAPDH	37	Mouse	Western blot	1:1000	Santa Cruz	Sc-32233
CD8	-[b]	Rabbit	Immunofluorescence	1:1000	Abcam	ab217344
	-[b]	Rat	Flow cytometry	1:200	BD Bioscience	553030
CD4	-[b]	Rabbit	Immunofluorescence	1:1000	Abcam	ab183685
	-[b]	Rat	Flow cytometry	1:200	BD Bioscience	553046
	-[b]	Mouse	Flow cytometry	1:200	BD Bioscience	561843
CD86	-[b]	Mouse	Immunofluorescence	1:1000	Genetex	GTX34569
	-[b]	Rat	Flow cytometry	1:200	BD Bioscience	742120
CD206	-[b]	Rabbit	Immunofluorescence	1:1000	Abcam	ab64693
	-[b]	Rat	Flow cytometry	1:200	BD Bioscience	565250
CD11b	-[b]	Rat	Flow cytometry	1:200	BD Bioscience	564454
Foxp3	-[b]	Rat	Flow cytometry	1:200	Invitrogen	12-5773-82
CD25	-[b]	Rat	Flow cytometry	1:200	BD Bioscience	562695
	-[b]	Mouse	Flow cytometry	1:200	BD Bioscience	565106
PD-1	-[b]	Hamster	Flow cytometry	1:200	BD Bioscience	562671
CTLA4	-[b]	Hamster	Flow cytometry	1:200	BD Bioscience	553720

CD3	-[b]	Mouse	Flow cytometry	1:200	BD Bioscience	563798
CD45	-[b]	Mouse	Flow cytometry	1:200	BD Bioscience	563204
PD-L1	-[b]	Rabbit	Immunohistochemistry	1:200	Cell Signaling	13684T

[a] ChIP: chromatin immunoprecipitation

[b] Molecular weight is not applicable to immunohistochemistry, immunofluorescence, or flow cytometry analysis of this antibody.
